# Use of Physiologically Based Kinetic Modeling to Predict Deoxynivalenol Metabolism and Its Role in Intestinal Inflammation and Bile Acid Kinetics in Humans

**DOI:** 10.1021/acs.jafc.3c07137

**Published:** 2023-12-22

**Authors:** Jingxuan Wang, Veronique de Bruijn, Ivonne M.C.M. Rietjens, Nynke I. Kramer, Hans Bouwmeester

**Affiliations:** Division of Toxicology, Wageningen University and Research, Stippeneng 4, 6708 WE, Wageningen, Netherlands

**Keywords:** bile acid malabsorption, deoxynivalenol, intestinal inflammation, physiologically based kinetic modeling, quantitative in vitro to in vivo extrapolation

## Abstract

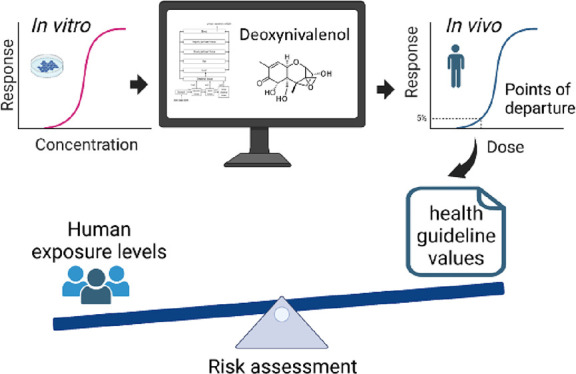

Current points of departure used to derive health-based guidance values for deoxynivalenol (DON) are based on studies in laboratory animals. Here, an animal-free testing approach was adopted in which a reverse dosimetry physiologically based kinetic (PBK) modeling is used to predict in vivo dose response curves for DON’s effects on intestinal pro-inflammatory cytokine secretion and intestinal bile acid reabsorption in humans from concentration–effect relationships for DON in vitro. The calculated doses for inducing a 5% added effect above the background level (ED_5_) of DON for increasing IL-1β secretion in intestinal tissue and for increasing the amounts in the colon lumen of glycochenodeoxycholic acid (GCDCA) were 246 and 36 μg/kg bw/day, respectively. These in vitro–in silico-derived ED_5_ values were compared to human dietary DON exposure levels, indicating that the risk of DON’s effects on these end points occurring in various human populations cannot be excluded. This in vitro–in silico approach provides a novel testing strategy for hazard and risk assessment without using laboratory animals.

## 1 Introduction

Deoxynivalenol (DON) is a foodborne trichothecene mycotoxin.^1^ Free-DON and its acetyl derivatives (3-Ac-DON and 15-Ac-DON) are produced by *Fusarium* fungi as secondary metabolites and are therefore regarded as unmodified mycotoxins. They can be modified by the plant infected by mycotoxin-producing fungi, resulting in the production of DON-3-glucoside.^1^ Four forms, namely, free-DON, 3-Ac-DON, 15-Ac-DON, and DON-3-glucoside, are the dominant forms of DON that contaminate cereal grains. In cereal grain-based food products, free-DON concentrations are on average five times higher than concentrations of DON-3-glucoside and an order of magnitude higher than concentrations of the acetyl DONs.^1^ In the intestinal lumen, acetyl DONs are largely deacetylated and DON-3-glucoside is cleaved, resulting in free DON.^2^ Thus, in a conservative approach to assess DON exposure applied by the European Food Safety Authority (EFSA),^1^ it is assumed that the DON derivatives are all metabolized to free DON before being absorbed.

Human exposure to DON occurs mainly via wheat and wheat-based products.^1^ The average dietary DON exposure varies from 0.2 to 14.5 μg/kg bw/day in different regions across the globe and is increasing in many regions due to climate change.^3,4^ To protect humans from the adverse effects of DON, a group provisional maximum tolerable daily intake (PMTDI) of 1 μg/kg bw per day was established for free-DON and its derivatives based on the reduced body weight gain in mice.^1^ Dietary DON exposure levels exceeding the PMTDI raises a food safety issue.^1,5^ The current hazard assessment of DON is based on data derived from studies using animal models.^2^ However, laboratory animals show notable differences in biokinetic activity and the metabolite pattern of DON compared to humans,^6^ raising the question of whether a hazard characterization using a so-called “new approach methodology” (NAM) based on human in vitro and in silico models would result in a different point of departure and/or a different health-based guidance value.

A NAM combining human cell-based in vitro studies with physiologically based kinetic (PBK) modeling for a mechanism-based risk assessment would remove species-dependent differences in the hazard assessment while at the same time minimizing animal testing for human risk assessment.^7^ PBK models describe the absorption, distribution, metabolism, and excretion (ADME) of a compound in time in a defined species using a set of mathematical equations based in physiological, exposure, and toxicokinetic parameters.^8^ PBK modeling has proven to be an efficient approach to translate in vitro toxicity data for different toxic end points to in vivo data that allow for the definition of points of departure to set health-based guidance values.^9,10^ In the current study, a PBK modeling-based in vitro–in silico approach was used to predict the dose–response behavior for selected adverse effects of DON in humans and derive the corresponding points of departure.

The intestinal tissue is the target organ for the adverse health effects of DON, and acute DON exposure can induce symptoms such as vomiting, abdominal pain, and diarrhea in humans.^1^ Chronic exposure of mice to DON at a human dietary level induced intestinal tissue damage due to intestinal inflammation.^11^ Central to the molecular and cellular key events triggered by DON are local inflammatory processes. The pro-inflammatory effects of DON can be studied in vitro using macrophages, which are the most abundant immune cells in the lamina propria of human intestine tissues involved in the local inflammatory processes. DON has shown to stimulate pro-inflammatory cytokine secretion in human immune THP-1 macrophages already at a low concentration of 0.5 μM.^12^ The pro-inflammatory effect in intestinal tissues triggered by DON was selected as the first adverse outcome for the quantitative in vitro-to-in vivo extrapolation (QIVIVE) analysis in this study. In addition, intestinal inflammation is associated with bile acid malabsorption in humans.^13^ Recent studies have shown that DON disrupts bile acid transport across intestinal epithelium Caco-2 cell layers.^14,15^ Bile acids are synthesized in the liver and secreted into the intestinal lumen via bile. Most bile acids are reabsorbed via the intestinal epithelium and are transported back to the liver.^16^ The DON-induced disruption of bile acid reabsorption in the ileum can be expected to increase the amount of bile acids in the colon lumen, which will eventually increase bile acid loss in the feces.^13^ Thus, bile acid malabsorption was selected as the second adverse outcome in this study.

To perform QIVIVE for DON-induced effects in in vitro cell models, a PBK model was developed that predicts the in vivo kinetics of DON in humans. Using the developed PBK model, the in vitro concentration-dependent effect of DON on IL-1β secretion by THP-1 cells was extrapolated to an in vivo dose–response curve by PBK modeling-based reverse dosimetry. The dose–response curve was analyzed to derive the effective dose of DON causing a 5% added effect above the background level (ED_5_) for IL-1β secretion in human intestinal tissue. Glycochenodeoxycholic acid (GCDCA) is the most abundant bile acid in the human bile acid pool.^17^ The effects of DON on GCDCA amounts in the colon lumen due to DON-mediated inhibition of ileal absorption were predicted by combining the PBK model for DON with a previously developed PBK model for GCDCA in human.^10^ From the predicted dose–response curve, the ED_5_ for a DON-mediated increase in the GCDCA amounts in the lumen of the human colon was derived. These in vitro–in silico-derived ED_5_ values were compared to the animal-derived point of departure and to human dietary DON exposure levels in various populations, including those in high wheat-consumption countries. Our study provides a novel testing strategy for hazard and risk assessment of DON with a minimal use of laboratory animals. The results contribute to the understanding of human health implications of DON contamination related to human exposure levels through cereal-based food products.

## 2 Materials and Methods

### 2.1 Cell Culture

Human immune THP-1 cells (passage numbers 18–35) were grown at 37 °C with 5% CO_2_ in RPMI1640 (Gibco BRL Breda, Netherlands), supplemented with 10% heat-inactivated fetal calf serum and 1% penicillin/streptomycin (Gibco BRL) (THP-1 culture medium). Human colon carcinoma Caco-2 cells (passage number 10–30) were grown at 37 °C with 5% CO_2_ in Minimum Essential Medium (MEM) (Gibco BRL), supplemented with 20% heat-inactivated fetal bovine serum, 1% pyruvate, and 1% penicillin/streptomycin/glutamine (Caco-2 culture medium).

### 2.2 In Vitro Pro-inflammatory Effect of DON on Immune THP-1 cells

The in vitro pro-inflammatory effect of DON on THP-1 macrophages was assessed by measuring the pro-inflammatory cytokine release following DON exposure. THP-1 cells were seeded at 1.8 × 10^5^ cells/well in a 12-well plate with 50 ng/mL phorbol-12-myristate-13-acetate (PMA) (Sigma-Aldrich, St Louis, MO, USA) for 24 h. After PMA induced differentiation, THP-1 macrophages were washed once and supplied with the culture medium for an additional 24 h. THP-1 macrophages were subsequently exposed to DON (0, 0.25, 0.5, 1, 2.5, and 5 μM) (Sigma-Aldrich) for 24 h in the culture medium. These concentrations of DON were shown to be noncytotoxic to THP-1 macrophages (Figure S1). The medium was collected, and the concentrations of IL-1β were quantified by an enzyme-linked immune-sorbent assay (ELISA) performed according to manufacturer’s instructions (Biolegend, San Diego, CA, USA).

### 2.3 In Vitro DON Exposure Reduced GCDCA Transport across Caco-2 Cell Layers

Caco-2 cells were seeded at 8.9 × 10^4^ cells/cm^2^ in 12-well polyethylene terephthalate membrane inserts (CellQART, Northeim, Germany) with a 0.4 μm pore size and maintained in culture for 14 days. The culture medium was changed every other day. Caco-2 cells cultured for 14 days were apically exposed to DON (0, 0.125, 0.25, 0.5, 1, and 2.5 μM) in the culture medium or for 7 days. The exposure medium was changed every other day. At day 21, the exposure medium was removed, and the Caco-2 cell layers were gently rinsed with Hank’s balanced salt solution (Gibco BRL) supplemented with 10 mM HEPES (transport medium). After a 30 min incubation in transport medium, 3.5 nmol of GCDCA (Sigma-Aldrich) in 0.5 mL of transport medium was added to the apical compartment. The amount of GCDCA in the basolateral compartment was measured after incubating for 120 min by LC/MS/MS. The Caco-2 apparent permeability coefficient (*P*_app_) of GCDCA was calculated and scaled to the absorption rate constant of GCDCA (ka _GCDCA_) in DON-primed Caco-2 cell layers using eqs 1–3
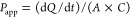
1
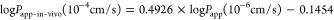
2
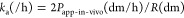
3where, in eq 1, d*Q*/d*t* (nmol/s) is the amount of GCDCA transported during the 120 min across the Caco-2 cell layer pre-exposed to different concentrations of DON, *A* is the surface area of the Caco-2 cell layer (1.12 cm^2^), and *C* is the initial concentration of GCDCA at the apical side (7 μM). To scale these in vitro Caco-2-based *P*_app_ values (calculated using eq 1) to human in vivo *P*_app_ values, eq 2(18) was applied. The absorption rate constant was subsequently calculated using eq 3.^19^ In eq 3, *R* is the average radius of the human intestine (0.25 dm).^20^ The absorption constant, *k*_a-__GCDCA_, of the control condition (0 μM DON) was set at 100%, and the reduction in the *k*_a_ due to inhibition of the transport by DON (0–5 μM) was expressed relative to the *k*_a_ of the control. The concentration–response data were fitted using a nonlinear regression curve fit, log (inhibitor or agonist) vs response-variable slope (four parameters) in GraphPad Prism 5, version 5.04 (GraphPad, San Diego California USA).

### 2.4 Quantifying GCDCA in the Transport Medium by LC/MS/MS

GCDCA in the transport medium was quantified using the LC/MS/MS Shimadzu 8045 System (Kyoto, Japan) as shown before.^14^ Aliquots of samples and standards (1 μL) were separated using a Phenomenex 00B-4475-AN column (50 mm × 2.1 mm × 1.7 μm × 100 Å, Kinetex C18) with Phenomenex AJ0–8782 (2 mm × 2.1 mm × 2.0 μm) as a guard column (Phenomenex, Torrance, CA, USA) at a column temperature of 40 °C. The flow rate of the mobile phases consisting of Milli-Q water with 0.01% formic acid (A) and methanol with 50% acetonitrile (B) was set at 0.4 mL/min: the starting gradient contained 30% B, which linearly increased to 70% B over 10 min then increased to 98% B at 11.0–18.0 min and finally was reduced to 30% at 19–25 min. The mass spectrometer (MS) used electrospray ionization in the negative-ion mode with the optimal electrospray ionization source parameters as follows: a nebulizer gas flow of 3 L/min, a heating gas and drying gas flow of 10 L/min, the interface temperature at 300 °C, the desolvation line temperature at 250 °C, and the heat block temperature at 400 °C. The multiple reaction-monitoring and selective ion-monitoring modes were used for quantification. Precursor and product ions were 448.3 and 74.0 *m*/*z.* The collision energy is 43 eV. Data were collected and processed using LabSolutions software, version 5.6 (Shimadzu).

### 2.5 Development of a PBK Model for DON in Humans

**Figure 1:**
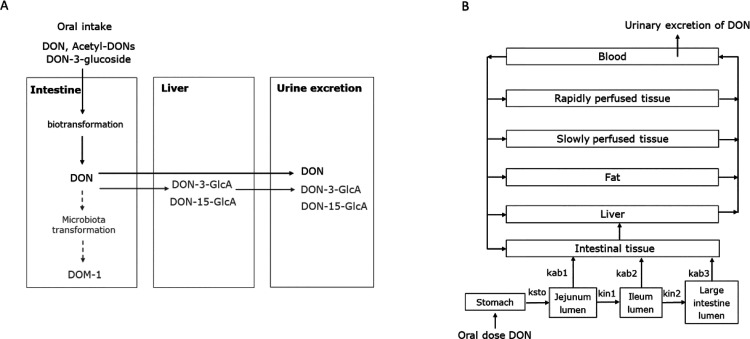
(A) Overview of the DON metabolism in humans. Solid arrows represent pathways observed in all humans, while dashed arrows indicate pathways specific to the DOM-1 producers. The black compound is included the current PBK model, whereas gray compounds are not included. (B) Schematic diagram of the PBK model of DON. The parameters ksto, kin1, and kin2 represent the transfer rates between the stomach, jejunum, and ileum, as determined in human studies.^26^ The parameters kab1, kab2, and kab3 are calculated using eqs 2 and 3 based on the *P*_app_ value of DON.^27^

Upon oral intake, DON derivatives are biotransformed to free-DON and are rapidly absorbed in the small intestine^1^ (Figure 1A). The absorbed free-DON is transported to the liver, where a large percentage is metabolized to DON-3-glucuronide (DON-3-GlcA) and DON-15-glucuronide (DON-15-GlcA).^6^ Up to 86.8% of the oral DON intake is excreted via urine with DON-15-GlcA being the main metabolite and free DON accounting for 20.1% of the excreted DON, and the fecal DON excretion is minimal.^21−23^ In addition, DON is detoxified by intestinal microbes resulting in the production of deepoxy-deoxynivalenol (DOM-1) in the intestines of DOM-1 producers, who represent less than 10% of the human population.^24^ DON-3/15-GlcA and DOM-1 are less abundant in the human intestine than free-DON, and they are less potent in inducing the pro-inflammatory response than free-DON.^1^ Moreover, these DON metabolites likely do not reduce bile acid transport because their molecular structure is larger than that of free-DON hampering their binding to ribosomes, which is the molecular initiating event of bile acid malabsorption.^25^ Thus, DON-3/15-GlcA and DOM-1 are not included in the current PBK modeling.

The schematic structure of the PBK model for DON is presented in Figure 1B. It includes separate compartments for segments of the gastrointestinal lumen, intestinal tissue, liver, fat, slowly perfused tissue, rapidly perfused tissue, and blood. To simulate the transfer of DON along the different segments of the gastrointestinal lumen and to account for the absorption across the intestinal epithelium lining these intestinal compartments, the gastrointestinal tract was included in the model with separate compartments for the stomach, jejunum lumen, ileum lumen, and large intestine lumen as done before.^26^

The transfer rate between each human gastrointestinal compartments was determined by Kimura and Higaki.^26^ Based on this work, the following rates were used: a stomach emptying rate ksto of 1.99/h, a transfer rate from jejunum to ileum kin1 of 2.17/h, and a transfer rate from ileum to large intestinal lumen kin2 of 0.25/h. The absorption of DON directly from stomach into the body is minimal.^28^ The stomach content rapidly passes through the pylorus into the jejunum lumen, bringing DON into contact with the absorptive surface of the intestine. Intestinal absorption of DON was described using a *P*_app_ value of 3.3 × 10^–6^ cm/s, which was obtained from the literature, reporting data from in vitro transport studies using cell layers of differentiated Caco-2 cells.^27^Equations 2 and 3 presented in Section 2.3 were applied to scale this in vitro *P*_app_ value to the in vivo absorption rate values kab1, ka2, and kab3, which are constant in the intestinal compartments.

To describe the systemic distribution of DON, the tissue/blood partition coefficients were predicted with the QIVIVE tool (Version 1.0) based on the Berezhkovskiy method and input parameters including an octanol–water partition coefficient (log*P*) of −0.71 (PubChem), a fraction unbound in plasma (*f*_up_) of 0.862, and a blood/plasma ratio (BPr) of 0.6523.^29^ The BPr and *f*_up_ of DON were predicted using the in silico Simcyp prediction tool (Certara, Sheffield, UK), resulting in an *f*_up_ that was in line with the *f*_up_ reported for plasma from a rat and sheep of approximately 0.9.^30,31^ Furthermore, DON is a neutral compound,^32^ and its predicted BPr was close to the default BPr of neutral compounds of 0.55.^33^ The tissue/blood partition coefficients values are shown in Table S1.

The kinetic constants for liver clearance of DON were derived from a literature reported study using in vitro incubations of DON with human liver microsomes.^34^ The study reported an in vitro clearance constant of DON of 0.008 mL/min/mg liver microsomal protein. This in vitro value was scaled to an in vivo value for the whole liver based on a human liver microsomal protein content of 32 mg of microsome protein/g of liver.^35^ The clearance of DON from blood was described by glomerular filtration and as such was included in the PBK model. The glomerular filtration rate is reported to be 1.8 mL/min/kg bw and was scaled to L/h as the excretion constant, assuming a body weight of 70 kg.^36^ The PBK model equations were run using Berkeley Madonna 10.2.8 (UC Berkeley, CA, USA), applying Rosenbrock’s algorithms for solving stiff systems. The full model code is available as Supporting Information Section S2. The human physiological parameters used in the PBK model are shown in Table S2.^37^

### 2.6 Evaluation of the PBK Model

The performance of the DON PBK model developed for humans was evaluated by comparing the predicted 24 h cumulative urinary free-DON amounts reported in the literature for human volunteers. Volunteers were exposed to a mixture of different forms of DON including free DON, acetyl DONs, and DON-3-glucoside via designed diets. The intake values of free DON, acetyl DONs, and DON-3-glucoside were reported in these human studies.^22,23^ The estimated intake values of DON were calculated based on the sum of all forms of DON. The detailed data and the calculations are shown in Table S3.

In addition, a local parameter sensitivity analysis was performed to identify parameters that influence the predicted *C*_max_ of DON in the intestinal venous plasma and ileum lumen. The method used for and the results of the sensitivity analysis are shown in Figure S2.

### 2.7 Quantitative Extrapolation

The in vitro concentration–response data for DON-induced IL-1β secretion were converted to in vivo dose–response curves using the developed PBK model for DON. For this purpose, the effective concentrations of unbound DON in the in vitro pro-inflammatory assay (*C*_in-vitro,IL-1β_ × *f*_ub-DON,in-vitro_) were set equal to the unbound in vivo maximum concentration of DON in the venous plasma of the intestine tissue (*C*_max-in-vivo,plasma_ × *f*_up_). The *f*_ub-DON,in-vitro_ indicates the fraction of unbound DON in the in vitro assay, which was set at 1 since DON did not bind to human serum albumin and the cytotoxicity of DON was not affected by adding up to 40 g/L human serum albumin to the exposure medium.^38^ The fraction of unbound DON in human plasma (*f*_up_) was 0.862, as indicated above. Overall, each concentration tested in the in vitro assay for IL-1β secretion was set equal to the *C*_max,in-vivo,intestine-plasma_ × *f*_up_ values. The developed PBK model was used to determine the corresponding oral dose levels. Thus, the entire in vitro concentration–response curve was translated to a predicted in vivo dose–response curve. The predicted dose–response curve was subsequently analyzed to define the ED_5_ for DON to stimulate IL-1β secretion using GraphPad as indicated in Section 2.3.

### 2.8 Prediction of the GCDCA Amounts in Human Colon Following DON Exposure

**Figure 2:**
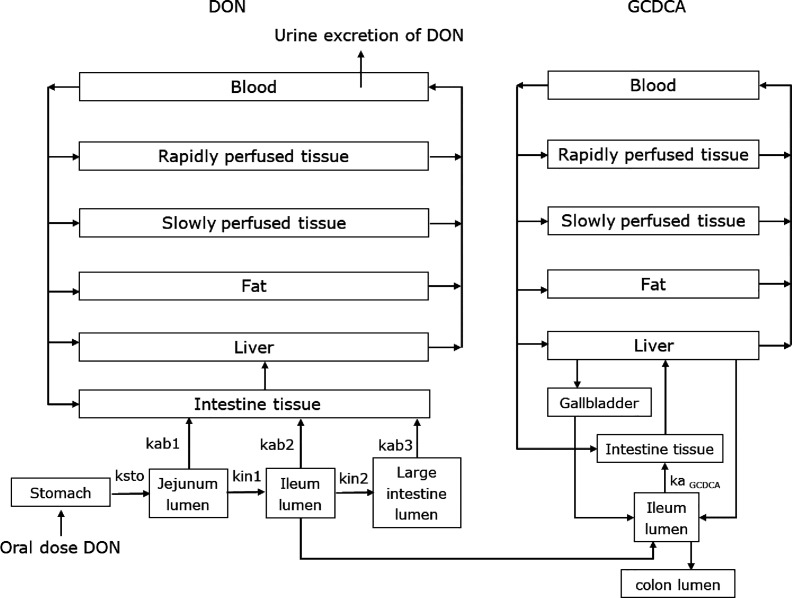
Schematic diagram of the PBK models of GCDCA and DON.

A schematic structure of the combined GCDCA and DON models is presented in Figure 2. The GCDCA model was adapted from a previous study performed by our group.^10^ In the GCDCA model, the intestinal reabsorption of GCDCA was described by the absorption rate constant *k*_a-__GCDCA_. In the previously developed GCDCA model,^10^ the *k*_a-__GCDCA_ was set at 1.047/h by fitting to available experimental data.^39^ Here, we included the *k*_a-GCDCA_ values in the presence of DON as the *k*_a-GCDCA_ values are influenced by different DON exposure concentrations. The *k*_a-__GCDCA_ was calculated based on the results from the Caco-2 transport study as *k*_a-__GCDCA_ = −231.5 + (0.9222 + 231.5)/(1 + 10^((7.360 – *C*_DON_)(−0.3966))) (see Section 2.3 and Figure 4C) where *C*_DON_ is the DON concentration in the ileum lumen. This equation was included in the previously developed PBK model for GCDCA to accommodate the reduction of GCDCA transport by DON present in the lumen of the ileum. In addition, the de novo GCDCA synthesis in liver was set as 0.78 × 60 (μmol/h/entire liver) (De Bruijn et al., 2022), and the fecal excretion of GCDCA was set equal to the de novo synthesis in the previous developed GCDCA model.^10^ In this study, the fecal excretion via the colon was set at 5% of the ileum GCDCA amounts and the de novo synthesis in liver was set equal to the fecal excretion.^40^ As for the DON PBK model, equations coding of the combined PBK model for DON and GCDCA were run using Berkeley Madonna, applying Rosenbrock’s algorithms for solving stiff systems. The full model code is presented in Supporting Information Section S3.

## Results

### Model Evaluation by Comparison of Predictions to Literature Data

**Figure 3:**
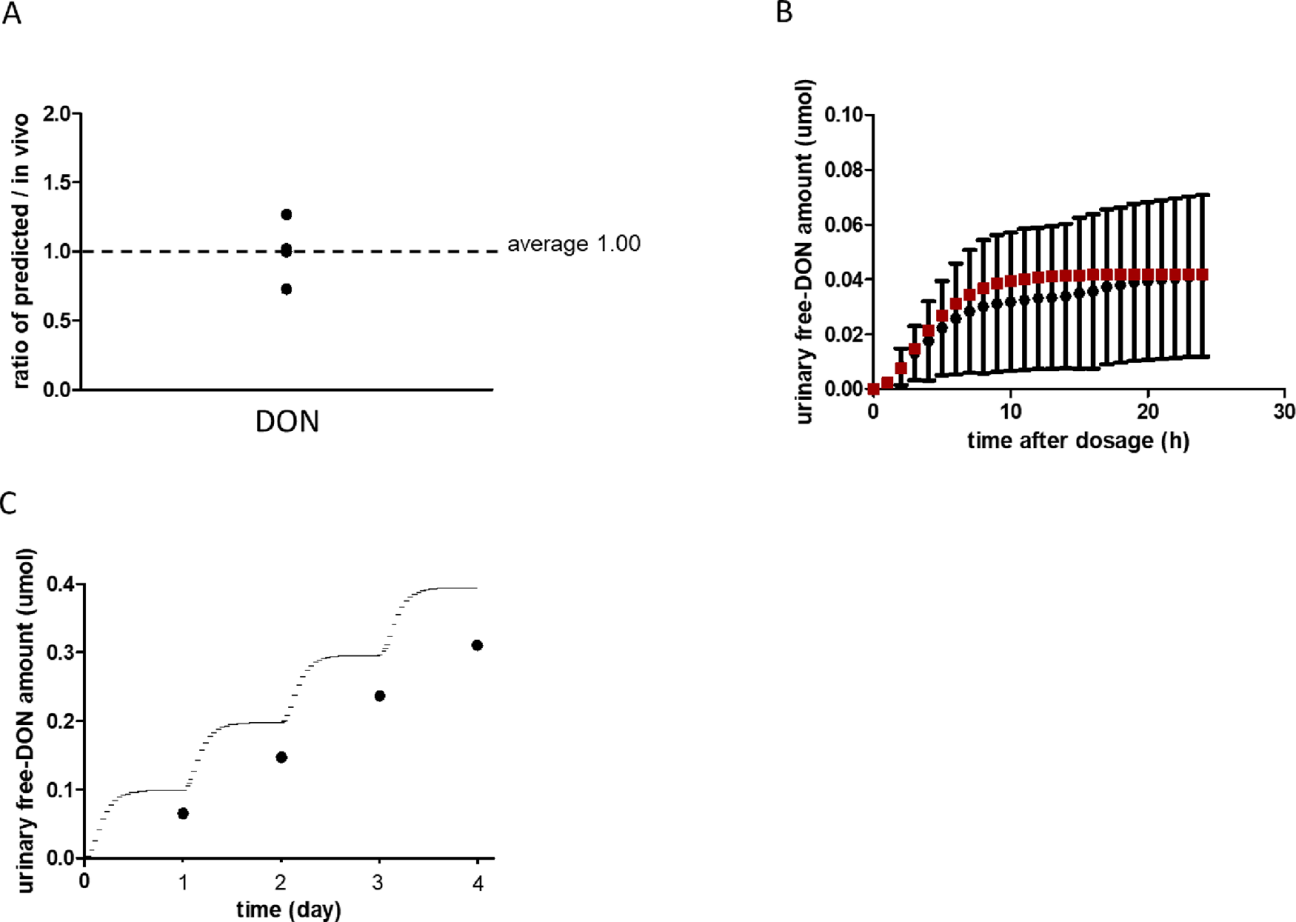
Comparison of reported and predicted urinary free-DON excretion by humans. (A) Ratio between the cumulative human urinary free-DON excretion in 24 h derived from the reported human data^22,23,41^ and the model predicted values. Each data point represents a separate ratio. (B) Time-dependent urinary free-DON excretion derived from the in vivo study and the predicted values. Solid circle: reported human in vivo data including the standard deviation;^41^ solid square: predicted time-dependent urinary free-DON excretion. (C) Urinary free-DON excretion derived from the in vivo study and the predicted values. Solid circle: reported human in vivo data;^23^ solid line: predicted time-dependent urinary free-DON excretion.

Urinary excretion of DON has long been used as a biomarker indicating DON exposure of humans.^31^ The availability of these data enabled the evaluation of the performance of the PBK model by comparison of the model-predicted urinary free-DON excretion to human urinary free-DON excretion data obtained from the literature. Table S3 shows the reported cumulative human urinary free-DON excretion data over 24 h after oral DON intake, which amounted to values from 1 to 2.90 μg/kg bw and also the PBK model-predicted human urinary free-DON excretions calculated by the PBK model using the corresponding DON intake values. The dose level of DON was calculated based on the sum of free DON, acetyl DONs, and DON-3-glucoside in the diet of these human studies^22,23^ (Table S3). The reported in vivo 24 h cumulative free-DON amounts in urine were calculated based on an average daily urinary volume of 2.42 L.^23,37^ Predictions were made using a mean human body weight of 70 kg (unless the body weight was mentioned in the study).^37^ The ratios between the predicted values and the values derived from the reported human data are 1.0, 0.73, 1.02, and 1.27 (Table 1). Thus, the model prediction is on average 1.00 ± 0.22 times the reported in vivo 24 h urinary excreted free-DON (Figure 3A).

**Table 1:** In Vivo and Predicted Cumulative Urinary Excretion of Free DON in Humans after 24 h of Oral Dosing of DON

DON intake (μg/kg BW)	in vivo urinary free-DON excretion for 24 h from literature (μmol)	urinary free-DON excretion for 24 h predicted in the current study (μmol)	ratio predicted/observed in vivo	remark
1	0.042^41^	0.042	1.00	single bolus; mean 24 h excretion value of 16 volunteers
2.90	0.148^22^	0.108	0.73	mean value of dietary DON intake in 1 day; mean 24 h excretion value of 83 volunteers (62.2 kg)
2.27	0.083^22^	0.085	1.02	median value of dietary DON intake in 1 day; median 24 h excretion value of 83 volunteers (62.2 kg)
2.75	0.078^23^	0.099	1.27	dietary DON intake in 1 day; mean 24 h excretion value of 1 volunteer (60 kg) during 4 days

Next, the PBK model was evaluated by comparing predicted time-dependent urinary free-DON excretion curves with in vivo kinetic data obtained for 24 h following a single oral dose of 1 μg/kg bw DON (Figure 3B).^41^ The results from the in vivo study indicated that free-DON was rapidly excreted within the first 6 h after oral intake.^41^ The PBK model adequately predicted this time-dependent urinary free-DON excretion for 24 h post dosing. Finally, the predicted urinary free-DON excretion was compared with in vivo data from one volunteer upon repeated dosing of 2.75 μg/kg bw/day DON over four days (Figure 3C).^23^ The cumulative urinary free-DON excretion amounted to 0.10, 0.20, 0.30, and 0.39 μmol at the end of days 1, 2, 3, and 4 for the predicted values and to 0.07, 0.15, 0.24, and 0.31 μmol for the in vivo determined values, respectively. The predictions were 1.5, 1.3, 1.2, or 1.3-fold higher than the in vivo value at the end of days 1, 2, 3, and 4, respectively. Together, these results indicate that the PBK model predicted urinary free-DON excretion in humans well.

### In Vitro Pro-inflammatory Effect of DON on Immune THP-1 Cells and Translation of the in Vitro Concentration–Response Data to an in Vivo Dose–Response Curve

**Figure 4:**
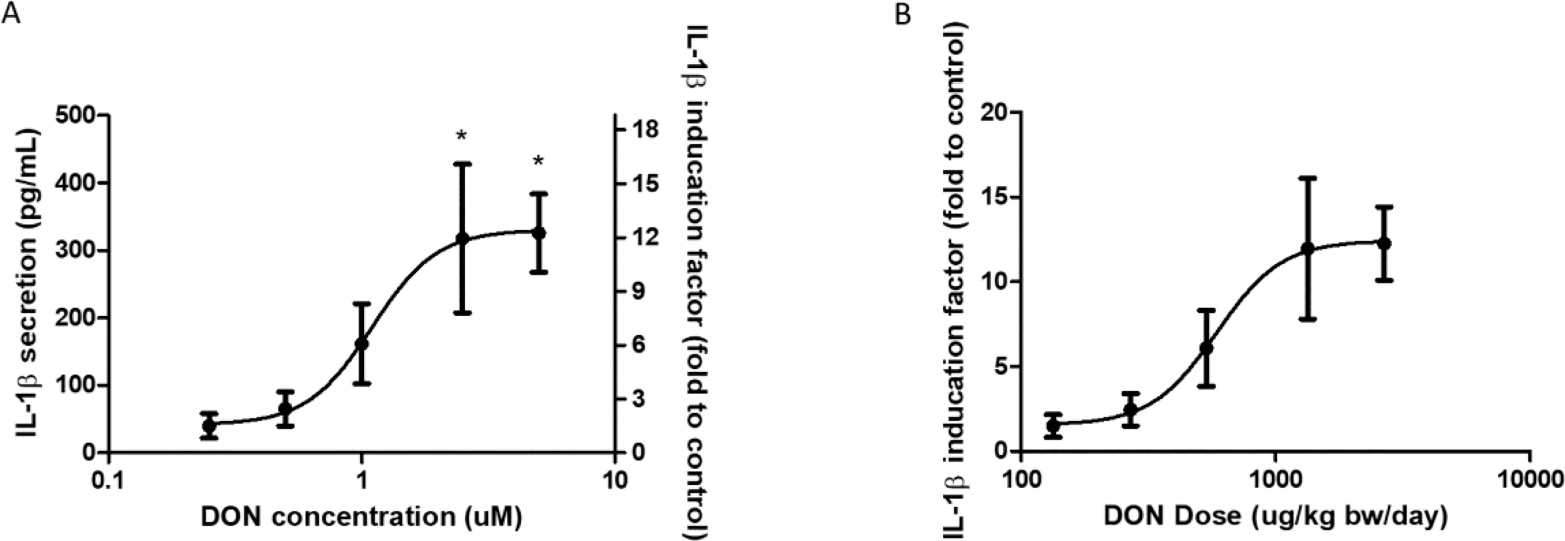
Effect of DON on in vitro and in vivo pro-inflammatory cytokine IL-1β secretion. (A) Concentration–response curves for the effect of DON on IL-1β secretion in THP-1 derived macrophages. The IL-1β concentrations are shown using the left *y* axis. The IL-1β concentration of the control condition (0 μM DON) was set at 1, and the induction factors of DON (0–5 μM) compared to control conditions are shown using the right *y* axis. Data were expressed as mean ± SD, *n* = 3. *: significantly different from the control group (*p* < 0.05). (B) Predicted in vivo dose–response curve for the DON-mediated induction of IL-1β secretion in the intestinal tissue, obtained by PBK modeling-based reverse dosimetry of the concentration–response curve in A.

Following absorption across the intestinal epithelial barrier, DON encounters immune cells in the intestinal tissue. Macrophages residing in the lamina propria of the intestinal tissue play an important role in the intestinal inflammatory regulation.^42^ The in vitro pro-inflammatory effect of DON on THP-1 macrophages was assessed by measuring pro-inflammatory cytokine IL-1β secretion. DON concentration-dependently increased the IL-1β secretion to 318 ± 191 or 326 ± 100 pg/mL (12.0 ± 7.2 fold or 12.3 ± 3.8 fold of control) following 2.5 or 5 μM DON exposure compared with 27 ± 13 pg/mL without DON exposure (Figure 4A). The in vitro concentration–response data for DON induced IL-1β secretion were converted to an in vivo dose–response curve using the developed PBK model for DON, taking differences in protein binding in the in vitro and in vivo situation into account (Figure 4B). The ED_5_ of DON for stimulating the IL-1β secretion in intestinal tissue, as derived from the predicted dose–response curve is 246 μg/kg bw/day.

### In Vitro DON Exposure Reduced GCDCA Transport across Caco-2 Cell Layers

**Figure 5:**
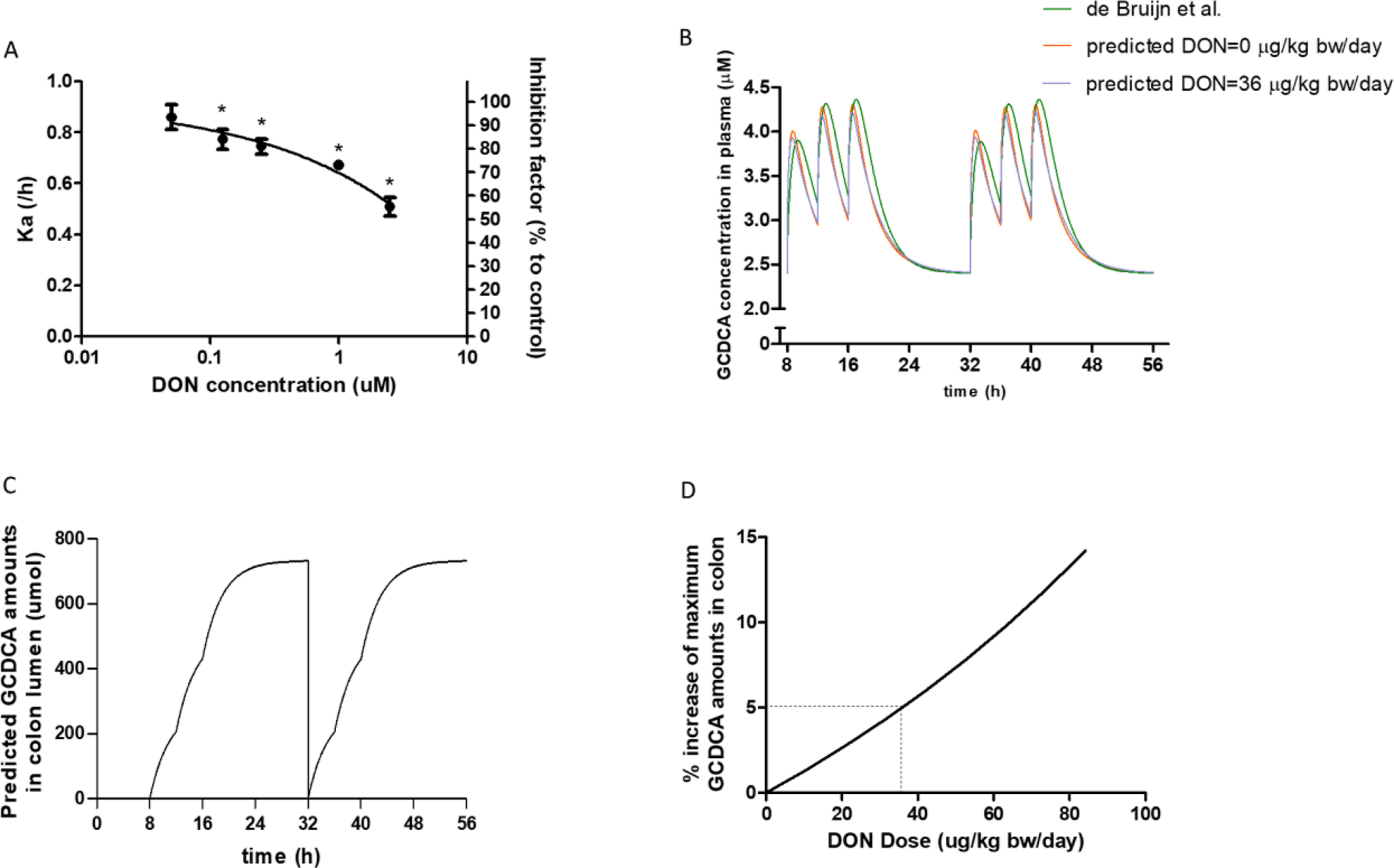
Effect of DON on in vitro and in vivo GCDCA absorption. (A) Concentration–response curve for the effect of DON on GCDCA transport across Caco-2 cell layers. The absorption rate constant (*k*_a_) of GCDCA is shown using the left *y* axis. The reduction factor of the *k*_a_ caused by DON (0–5 μM) compared to control conditions (0 μM DON) is shown using the right *y* axis. Data were expressed as mean ± SD, *n* = 3. *: significantly different from the control group (*p* < 0.05). (B) Predicted GCDCA concentrations in human plasma for two days at a DON exposure concentration of 0 or 36 μg/kg bw/day (three times 12 μg/kg bw/meal) and the prediction from a previously reported PBK model for GCDCA.^10^ (C) Predicted GCDCA amounts in the lumen of the colon for two days at a DON exposure concentration set at 0 μg/kg bw/day. (D) Predicted % increase in the 24 h maximum GCDCA amounts in the lumen of the colon following DON exposure (0–84 μg/kg bw/day). The daily DON exposure dose was assumed to be equally distributed over three meals. DON exposure and GCDCA gallbladder secretion was assumed to occur during the meals (exposed at 8:00, 12:00, and 16:00 h) and stopped at night.

The transport of GCDCA across Caco-2 cell layers was studied following 0–2.5 μM DON exposure as DON does not affect the Caco-2 barrier integrity at concentrations up to 2.5 μM.^14^ DON-pre-exposed Caco-2 cells were apically exposed to GCDCA, and the transport of GCDCA from the apical to the basolateral compartment was determined. The *k*_a-GCDCA_ dose-dependently decreased from 0.92 ± 0.01/h (without DON exposure) to 0.77 ± 0.07, 0.75 ± 0.05, 0.67 ± 0.01, and 0.51 ± 0.06/h following 0.125, 0.25, 1.0, and 2.5 μM DON pre-exposure (Figure 5A). The reduction in GCDCA transport was statistically significant (*p* < 0.05) at all tested DON concentrations and amounted to 84 ± 7%, 81 ± 6%, 73 ± 1%, and 55 ± 7% of the control for 0.125, 0.25, 1.0, and 2.5 μM DON pre-exposed cells. An equation fitting the concentration–response curve for the DON induced reduction of GCDCA transport across Caco-2 cell layers is *k*_a-GCDCA_= −231.5 + (0.9222 + 231.5)/(1 + 10^((7.360 – *C*_DON_)(−0.3966))).

### Prediction of the GCDCA Amounts in Human Colon Following DON Exposure

A first check of the current-combined PBK model of GCDCA and DON was performed by comparing the predicted time-dependent GCDCA plasma concentration profile in the absence of DON (exposure concentration set at 0 μg/kg bw/day) with the plasma concentration-versus-time profile predicted by the previously reported GCDCA model (Figure 5B). In this modeling, it was assumed that the subject was fasted overnight and meals were simulated at 8:00, 12:00, and 16:00 h. Upon meal ingestion, the gall bladder contracted, resulting in a peak in the systemic GCDCA concentration in plasma. The predicted systemic maximum GCDCA concentration in plasma was 4.31 μM, which is close to 4.36 μM of the maximum plasma GCDCA concentration predicted by the previously developed PBK model of GCDCA.^10^ The minimal difference is mainly due to the difference in the *k*_a_ values and in the hepatic GCDCA de novo synthesis values between these two models as shown in Section 2.8.

Bile acids that escape reabsorption in the ileum will enter the colon lumen and are eventually lost in the feces.^16^ To predict the GCDCA amounts in the colon lumen following DON exposure, it was assumed that, at 8:00 each day, the amount of GCDCA in the colon lumen equals zero due to fecal excretion. Figure 5C shows the predicted subsequent increase in the amount of GCDCA in the colon lumen over time for two days in the absence of DON exposure. The maximum GCDCA amount in the colon lumen reaches 733 μmol 24 h after 8:00 after which the GCDCA amount in the colon lumen returns to zero following fecal excretion. When modeling the effect of DON exposure, it was assumed that the daily DON exposure was equally distributed over three meals with meals simulated at 8:00, 12:00, and 16:00. The predicted % increase of the maximum GCDCA amounts in the colon lumen at different daily accumulated DON exposure levels (assumed to be equally distributed over three meals) is shown in Figure 5D. The DON exposure predicted to result in a 5% increase of the maximum GCDCA amount in the colon lumen was 36 μg/kg bw/day (three times of 12 μg/kg bw/meal). Increased GCDCA amounts in the colon lumen are accompanied by decreased GCDCA levels in plasma. Next, we predicted the GCDCA plasma levels upon DON exposure at 36 μg/kg bw/day (three times of 12 μg/kg bw/meal). The predicted systemic maximum plasma GCDCA concentrations decreased to 4.19 μM, which is a 2.8% reduction from that in the absence of DON exposure (Figure 5B).

### Comparison of the Derived ED_5_ Values Predicted for the Effect of DON on Pro-inflammatory Cytokine IL-1β Secretion and GCDCA Accumulation in the Colon Lumen to Dietary DON Exposure Levels

In Figure 5, the predicted ED_5_ values of DON for stimulating IL-1β secretion in the intestinal tissue (246 μg/kg bw/day) and for increasing the maximum GCDCA amount in the colon lumen (36 μg/kg bw/day) were compared to the BMD_5_ (benchmark dose causing 5% extra effect above background level) of 190 μg/kg bw/day for reducing body weight gain in mice.^1^ This reveals that the ED_5_ for IL-1β secretion is in line with the BMD_5_ for reduced body weight gain, whereas the bile acid accumulation appears to be more sensitive.

**Figure 6:**
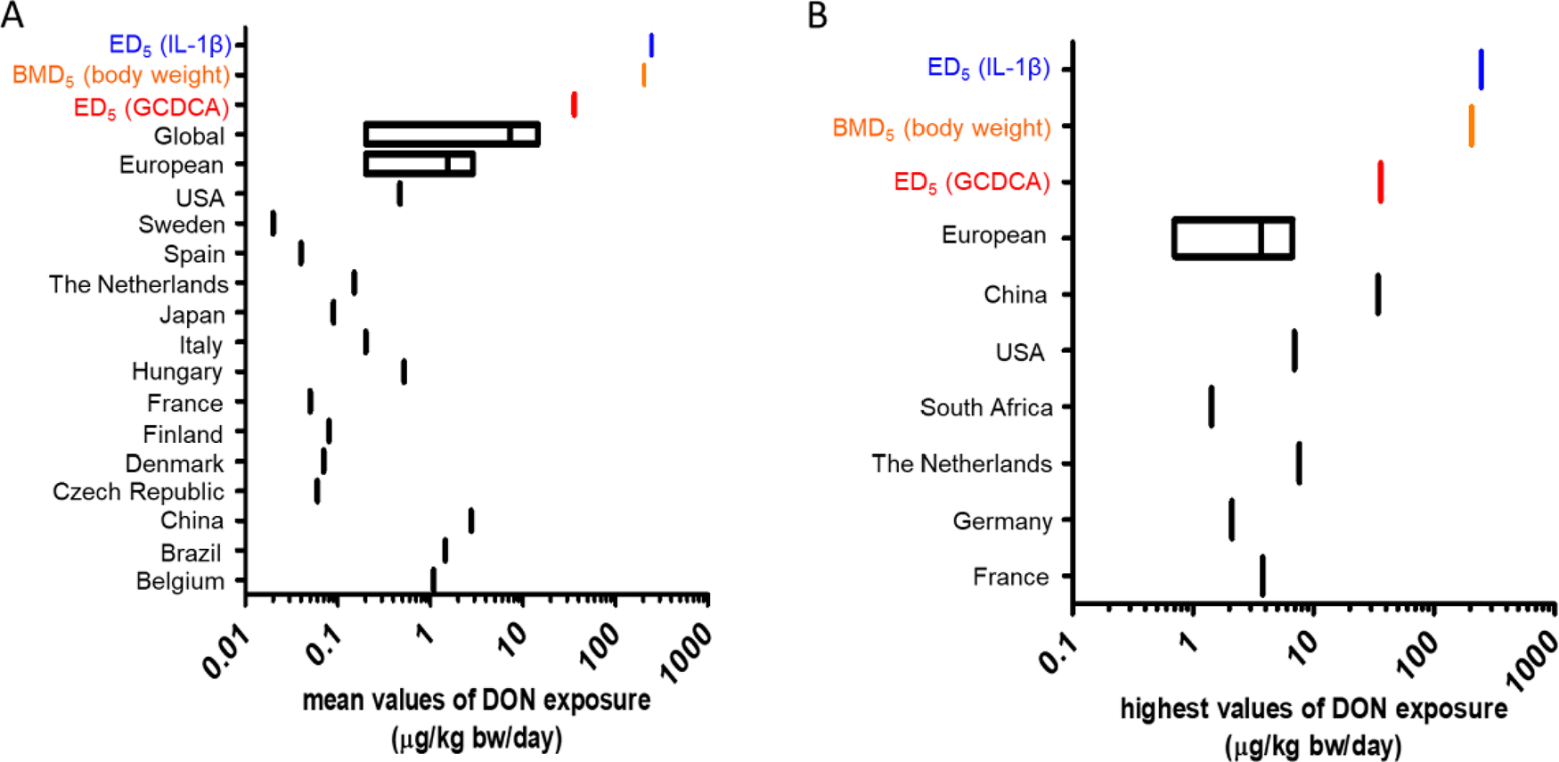
Comparison of the derived ED_5_values with dietary DON exposure levels in different countries. Comparison of the ED_5_ values for increasing IL-1β secretion in the intestinal tissue and for GCDCA amounts in the colon lumen with (A) the mean values of dietary DON exposure and with (B) the highest values of dietary DON exposure in different high wheat-consumption countries.^5^ The ED_5_ for IL-1β secretion in the intestinal tissue is defined to be 246 μg/kg bw/day (blue vertical bars). The ED_5_ of DON for increasing GCDCA in the colon lumen is defined to be 36 μg/kg of bw/day (red vertical bars). The BMD_5_ for reducing body weight gain in mice is 190 μg/kg bw/day (orange vertical bars).

Next, these derived ED_5_ and BMD_5_ values were compared with the available human dietary DON exposure data. JECFA estimated that the mean dietary DON exposure varies from 0.2 to 14.5 μg/kg bw/day in different regions across the globe.^3^ EFSA reported that the mean dietary DON exposure ranged from 0.2 to 2.9 μg/kg bw/day in European countries (EFSA, 2017). These mean dietary DON exposure values are 2–180, 17–1230, and 13–950-fold lower than the ED_5_ of DON for increasing the maximum GCDCA amount in the colon lumen, the ED_5_ for increasing IL-1β secretion, and the BMD_5_ for reducing body weight gain, respectively (Figure 6A). Recently, human dietary DON exposure levels were estimated by Chen et al. based on wheat consumption data and DON contamination data from different high wheat-consumption countries.^5^ Among all these countries, China (2.77 μg/kg bw/day), Brazil (1.45 μg/kg bw/day), and Belgium (1.08 μg/kg bw/day) are the three countries with the highest intake of DON from the diet. These exposure levels are 33, 228, and 176-fold below the ED_5_ of DON for increasing the maximum GCDCA amount in the colon lumen, the ED_5_ for increasing IL-1β secretion and the BMD_5_ for reduced body weight gain, respectively (Figure 6A). For the other countries, the mean values of DON exposure ranged from 0.02 μg/kg of bw/day in Sweden to 0.52 μg/kg of bw/day in Hungary. All of these mean values of DON exposure are 69–1800-fold lower than the ED_5_ of DON for the increased maximum GCDCA amount in the colon lumen and at least 473 and 365-fold below the ED_5_ for increasing IL-1β secretion in the intestine tissue and the BMD_5_ for reducing body weight gain (Figure 6A).

The highest DON exposure data representing the 95th percentiles for the respective populations vary from 0.7 to 6.7 μg/kg bw/day in European countries (EFSA, 2017). These highest exposure values are 5–51-fold lower than the ED_5_ for increasing GCDCA amounts in the lumen of the human colon, 37–351-fold lower than the ED_5_ for increasing IL-1β secretion in the intestinal tissue, and 28–271-fold lower than the BMD_5_ for the reduction in body weight gain. Chen et al. reported the highest DON exposure data for the 97.5th percentile range from 1.42 μg/kg bw/day in South Africa to 34.25 μg/kg bw/day in China,^5^ which are all more than 300-fold lower than the ED_5_ for increasing GCDCA amounts in the lumen of the human colon, the ED_5_ for increasing IL-1β secretion in the intestinal tissue, and the BMD_5_ for reduction in body weight gain (Figure 6B).

## Discussion

The aim of the present study was to investigate at what dose levels DON would be expected to stimulate IL-1β secretion in human intestinal tissue and to increase the GCDCA amounts in the lumen of human colon in vivo by using an in vitro–in silico testing strategy. For this, a PBK model was developed to predict in vivo kinetics of DON in humans, which in the next step was combined with a previously developed and validated PBK model for GCDCA. DON concentration-dependently stimulated IL-1β secretion in THP-1 cells. This in vitro concentration–response curve was converted to an in vivo dose–response curve by using PBK modeling-based reverse dosimetry. The ED_5_ of DON for increasing IL-1β secretion derived from this predicted dose–response curve amounted to 246 μg/kg bw/day. In addition, DON concentration-dependently decreased the level of GCDCA transport across Caco-2 cell layers. The GCDCA amounts in the lumen of human colon following DON exposure were predicted by using the combined PBK model of DON and GCDCA, inserting a reduced *k*_a-__GCDCA_ for uptake of GCDCA from the ileum lumen to the intestinal tissue into the PBK model at the dose level of DON that matched the in vitro concentration in the Caco-2 system where the reduced uptake was quantified. The ED_5_ of DON for increasing the GCDCA amounts in the lumen of human colon derived from the predicted dose–response curve was 36 μg/kg bw/day. The ED_5_ values of 246 μg/kg bw/day and 36 μg/kg bw/day thus obtained were compared to the BMD_5_ of 190 μg/kg bw/day for reducing body weight gain in mice (EFSA, 2017) and the human dietary DON exposure levels in various populations.^1,5^

To enable the prediction of the in vivo kinetics of DON in humans, a PBK model for DON was developed. Urinary excretion of DON has long been used as a biomarker for DON intake in humans.^31^ Our PBK model-based predictions matched the in vivo data well with the predicted urinary DON excretion being only 1.00, 0.73, 1.02, and 1.27 times different from the in vivo urinary DON excretion data available in the literature.^22,23,41^ However, Rodríguez-Carrasco et al. reported that an estimated dietary DON intake of 49.2 μg/day resulted in urinary DON excretion of 35.2 μg for 24 h in a 72 kg volunteer.^43^ In this case, the PBK model prediction was 0.3 times this in vivo data, which is somewhat outside the proposed range of 0.5 to 2 times the in vivo data for an acceptable match (OECD 2021). However, the results of this human study reported that 71.5% of the ingested DON was excreted as free DON during 24 h in urine.^43^ Numerous studies have shown that, although urinary excretion of DON and its glucuronide-conjugated metabolites accounts for up to 86.8% of the oral dosing after 24 h in human, only 20.1% DON is excreted as free DON.^21−23^ Therefore, the seemingly too low predictions may be correct as the excretion of DON in the conjugated form was not taken into account in the in vivo data. Correcting the in vivo data assuming that the urinary free DON excretion amounts to only 20.1% of the total 86.8% of DON excreted in urine, the excreted amount changes from 35.2 to 7.08 μg so that the prediction is only 1.2-fold higher than the corrected in vivo data.

Using the validated PBK model for DON and the PBK modeling-facilitated QIVIVE, an ED_5_ of 246 μg/kg of bw/day was derived for DON to increase IL-1β secretion in intestinal tissue. In addition to IL-1β, more immune parameters are involved in the in vitro pro-inflammatory effect of DON on human immune cells.^44^ It has been reported that DON concentration-dependently increased IL-8, TNFα, and IL-6 secretion in human U-937 macrophages^45^ and increased IL-2 secretion in human primary lymphocytes.^46^ By using the same PBK modeling-facilitated reverse dosimetry, we also converted these in vitro concentration–response curves to in vivo dose–response curves. The ED_5_ for increasing IL-8, IL-2, TNFα, and IL-6 secretion in the intestinal tissue was predicted to be 57, 70, 250, and 1070 μg/kg bw/day, respectively (Figure S3). The ED_5_ values for increased excretion of TNFα and IL-1β are relatively high, whereas the ones for IL-8 and IL-2 are lower and DON stimulated IL-6 excretion is predicted to occur with the highest ED_5_ in the human intestinal tissue. Since the kinetics of DON are similar for all the QIVIVEs, the differences in the sensitivity of the cytokine responses toward DON are more likely due to differences in the in vitro effects of DON on the production of these pro-inflammatory cytokines. Upon DON exposure, the MAPK pathways were activated, which mediate the activation of downstream transcription factors, such as NF-kB, AP-1, and CREB, and increase the production of pro-inflammatory cytokines in immune cells.^44^ IL-8 excretion was the most sensitive cytokine release-related end point following DON exposure, which is consistent with its function in the early stages of the immune response.^44^ IL-2 production in human primary lymphocytes was more sensitive than TNFα and IL-1β production in human macrophages. This may be due to the different immune cell models. Human primary lymphocytes include T cells, B cells, and Natural Killer (NK) cells. In a mixture of different types of human immune cells, the cells can potentiate each other, which may enlarge the inflammatory signal upon DON exposure.^44,46^ In human macrophages following DON exposure, the production of IL-6 was not as sensitive as that of other cytokines.^45^ This is in line with other studies reporting that the increase of IL-6 production was not significant in human lymphocytes following DON exposure.^46^

Furthermore, DON inhibited GCDCA transport across Caco-2 cell layers, increasing the GCDCA amount in the lumen of the human colon and decreasing the GCDCA plasma levels. The maximum GCDCA amount is predicted to be 733 μmol in the colon lumen without DON exposure, which is comparable to the reported data that indicate that healthy adults consuming a mixed western diet excrete up to 1 mmol of bile acids in feces each day.^47^ The predicted ED_5_ of DON for increasing GCDCA amounts in the colon lumen by 5% was 36 μg/kg of body weight (bw)/day, resulting in a 2.8% reduction in the maximum GCDCA concentration in human plasma. The reduced GCDCA plasma levels will stimulate the hepatic de novo synthesis of GCDCA via a negative feedback pathway,^16^ which will further increase the amount of GCDCA that is secreted into the ileum lumen and eventually lost into the colon lumen upon DON exposure. This feedback pathway is not included in the current PBK model of GCDCA. Thus, the DON-induced increase in the amount of colonic GCDCA is likely underestimated. Furthermore, the individual variations in human colonic bile acid concentrations can be large.^10,47^ Further studies are needed to investigate the potential adverse health effects associated with a 5% increase in GCDCA levels in the human colon lumen.

The ED_5_ of DON for increasing GCDCA amounts in the human colon (36 μg/kg bw/day) is lower than that for increasing pro-inflammatory cytokine secretion in the intestinal tissue (56–1070 μg/kg bw/day) and also lower than the animal-based BMD_5_ for reducing body weight gain in mice (190 μg/kg bw/day). This suggests that the GCDCA malabsorption is a more sensitive end point in humans following DON exposure than reduction in body weight or effects on pro-inflammatory cytokine secretion in intestinal tissue. Upon reaching the colon, high amounts of GCDCA stimulate electrolyte and water secretion, resulting in diarrhea in humans.^13^ Although the mode of action is not fully understood, diarrhea is one of the adverse outcomes following DON exposure in human outbreaks.^2^ Diarrhea is an important cause of malnutrition, which leads to body weight loss in adults.^13^ Thus, it could be speculated that the DON-induced bile acid malabsorption resulting in elevated bile acid levels in the colon may contribute to the DON induced diarrhea and associated body weight loss. Moreover, high amounts of bile acids reduce intestinal integrity and increase intestinal permeability in the colonic crypts of pig^48^ and in the small intestine of rabbit.^49^ Reduced intestinal integrity is related to pro-inflammatory cytokine production and inflammatory bowel disease in human intestine.^16^ Thus, the pro-inflammatory cytokine production in the intestinal tissue and the reduced body weight gain could be triggered by bile acid malabsorption in the human intestine, which, based on the results of the present study, appears to be a more sensitive end point upon DON exposure in humans than effects on body weight or pro-inflammatory cytokine secretion.

Next, ED_5_ and animal-based BMD_5_ were compared to human dietary DON exposure data. To enable an interpretation of this comparison, we first defined a margin of exposure value that would not raise a safety concern. To this end, a suitable point of departure that would be equivalent to a BMDL_5_, (the lower confidence limit of the BMD_5_), was assumed to be threefold lower than the BMD_5_ or ED_5_. This assumption was needed given that the dose response curves predicted for the effects of DON on IL-1β excretion and increasing GCDCA amounts in the human colon were unsuitable for BMD modeling, so a BMDL_5_ could not be calculated. This assumption seems realistic given that the actual difference between the BMDL_5_ for reduction in weight gain of 110 μg/kg bw/day is 1.7-fold lower than the corresponding BMD_5_ of 190 μg/kg bw/day.^1^ Together with the default uncertainty factors of 10 for interspecies and intraspecies differences each,^50^ this results in a margin of 300 to define a safe margin between the BMD_5_ or ED_5_ values and an EDI value. One could argue that taking into account an uncertainty factor of 10 for intraspecies differences would not be needed when the point of departure is obtained using human data from a human in vitro assay and human PBK models. However, considering that the definition of an ED_5_ using an in vitro and in silico-based NAM brings extra uncertainties, it seems prudent to maintain a default uncertainty factor of 100.^51^ Taking all of this together, it was assumed that a margin of 300 would not raise safety concerns when comparing the ED_5_ and BMD_5_ values to the human dietary DON exposure data.

The margin of exposure between the mean values of dietary DON exposure levels and the ED_5_ for increasing the maximum GCDCA amount in the colon lumen was found to be below this value of 300 for EDI from most countries, indicating a potential safety concern. Among all the countries, China, Brazil, and Belgium showed the highest DON exposure levels. The margins of safety in these three countries were below 300 for both the ED_5_ for increasing pro-inflammatory IL-1β secretion in intestinal tissue and the BMD_5_ for reducing body weight gain. This indicates that the effect of DON on pro-inflammatory IL-1β secretion in intestinal tissue and on body weight gain cannot be fully excluded. Furthermore, the highest DON exposure levels in all countries were below or close to 300 compared to the ED_5_ for increasing the GCDCA amount in the colon lumen, the ED_5_ for increasing IL-1β secretion in intestinal tissue and the BMD_5_ for reducing body weight gain, indicating such effects cannot be fully excluded for high level consumers. When using the BMDL_5_ of 110 mg/kg bw/day instead of the BMD_5_ for the reduction in body weight in mice, some EDI values resulted in margins that were lower than 100, thereby corroborating that, at the current levels of intake, the effects of DON on body weight gain cannot be fully excluded.

In conclusion, the present study shows a proof-of-principle for an in vitro–in silico based testing strategy to predict in vivo kinetics of DON and characterize its role for intestinal pro-inflammatory cytokine secretion and bile acid malabsorption in humans. The results obtained suggest bile acid malabsorption to be a more sensitive end point for DON exposure than a reduction in body weight gain and also that an effect of DON on these end points cannot be fully excluded in various populations. This in vitro–in silico approach provides a novel testing strategy without using laboratory animals for hazard and risk assessment.

## Supporting Information Available

Additional experimental details, parameters used for the PBK model of DON in humans, calculation of estimated intake values of DON in human studies, sensitivity analysis, effect of DON on pro-inflammatory cytokines production and reverse dosimetry; PBK code of DON; and PBK code of DON and GCDCA (PDF)

## Abbreviations

DON: deoxynivalenol
PMTDI: provisional maximum tolerable daily intake
PBK: physiologically based kinetics
QIVIVE: quantitative in vitro to in vivo extrapolation
GCDCA: glycochenodeoxycholic acid
BMD: benchmark dose
*P*_app_: apparent permeability coefficients
LogP: octanol–water partition coefficient
BW: body weight
ELISA: enzyme-linked immune-sorbent assay
PMA: phorbol-12-myristate-13-acetate
EFSA: European Food Safety Authority
