# Supplementary material for: Use of Physiologically Based Kinetic Modeling to Predict Deoxynivalenol Metabolism and Its Role in Intestinal Inflammation and Bile Acid Kinetics in Humans
Source: J Agric Food Chem. 2023 Dec 22;72(1):761–72. doi: 10.1021/acs.jafc.3c07137 (PMC10786035; doi:10.1021/acs.jafc.3c07137)
Supplement: Supplementary file 1 — jf3c07137_si_001.pdf [file jf3c07137_si_001.pdf]

Use of physiologically based kinetic modeling to predict deoxynivalenol metabolism and its role in intestinal inflammation and bile acid kinetics in humans

Jingxuan Wang[1]*, Veronique de Bruijn[1], Ivonne Magdalena Catharina Maria Rietjens[1], Nynke I. Kramer[1], Hans Bouwmeester[1]

[1]Division of Toxicology, Wageningen University and Research, Stippeneng 4, 6708 WE Wageningen, The Netherlands

*Corresponding author: Jingxuan Wang: Jingxuan.wang@wur.nl

Supplementary material 1

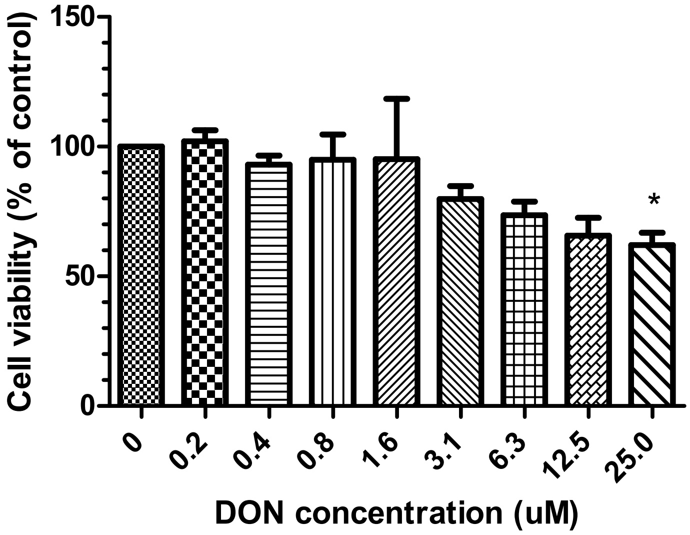

Fig.S1. The viability of THP-1 macrophages following exposure to different concentrations of DON (0-25 μM) determined by the WST-1 assay. Data were expressed as mean ± SD, n = 3. Statistical analysis was performed by ANOVA followed by the Dunnett test. *Significantly different from the control group ($p < 0.05$).

Table S1 Tissue/blood partition coefficients used in the human PBK model for DON

Tissue/blood partition coefficient	Liver	Rapidly perfused tissue	Slowly perfused tissue	Fat	Intestine tissue
DON	1.17	1.21	0.99	0.28	1.12

Table S2. Parameters used for the PBK model of DON in human [1].

Physiological parameters	Human	Physiological parameters	Human
Body weight (BW; kg)	70	Cardiac output (QC; L/h)	15*BW^0.74
Percentage of body weight		*Percentage of cardiac output*	
Liver	0.026	Liver	0.046
Fat	0.214	Fat	0.052
Rapidly perfused tissue	0.055	Rapidly perfused tissue	0.473
Slowly perfused tissue	0.603	Slowly perfused tissue	0.248

Intestine tissue	0.009	Intestine tissue	0.181
Blood	0.079		

Table S3. Estimated intake values of DON from designed diets in human studies. The estimated intake values of DON was calculated based on the sum of free-DON, 3-Ac-DON, 15-Ac-DON and DON-3-glucoside in the processed food.

DON Intake (μg/kg BW)	Free-DON (μg/kg BW)	3-Ac-DON (μg/kg BW)	15-Ac-DON (μg/kg BW)	DON-3-glucoside (μg/kg BW)
**1 [2]**	1	-	-	-
**2.90 [3]**	2.57	0.008	0.014	0.31
**2.27 [3]**	2.04	0.023	0.046	0.16
**2.75 [4]**	2.30	0.33	-	0.12

Sensitivity analysis

A local parameter sensitivity analysis was performed to identify parameters that influence the predicted C_{max} of DON in intestinal venous plasma and ileum lumen. The normalized sensitivity coefficient (SC) was calculated with the following equation [5]:

SC = (C'-C)/(P'-P) × P/C

where C is the initial value of the model output, C' is the modified model output value resulting in a 5% increase of the C. P is the initial parameter value, P' is the parameter value with a 5% increase of P. The analysis was conducted with an oral dose of 1 μg/kg bw based on PMTDI of DON. Parameters resulting in an normalized SC (absolute value) > 0.1 are considered to affect the model output [6]. From the results presented in **Fig.S2**, it follows that the predicted C_{max} of DON in the intestine venous plasma is most sensitive to the blood/plasma ratio of DON (RDON), the fraction of blood flow to intestine tissue (QIc) and the body weight (BW). The predicted C_{max} of DON in ileum lumen is most sensitive to the average diameter of small intestine (D), the length of ileum (L2) and the body weight (BW) (**Fig.S2**).

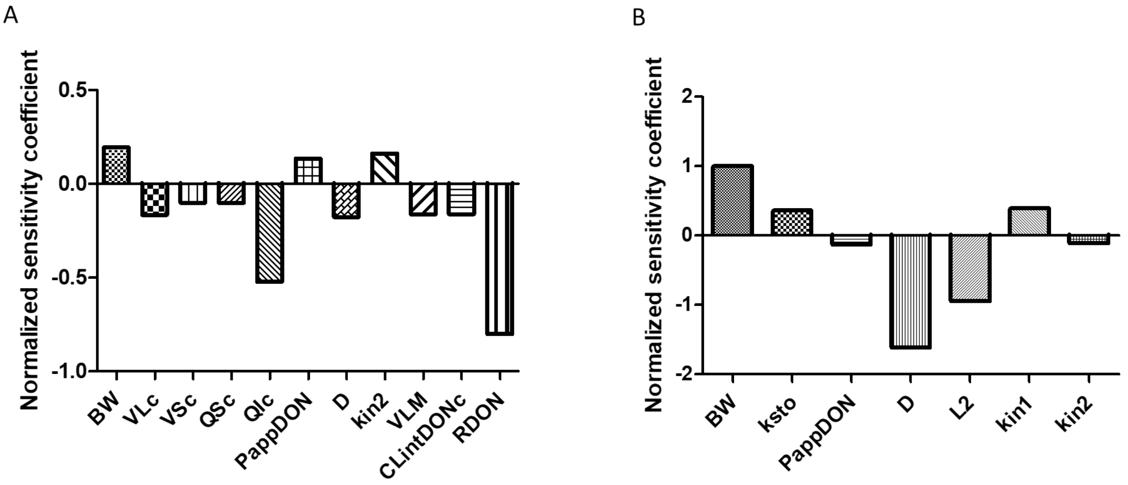

Fig. S2. Sensitivity analysis of the predicted DON concentration at oral dose of 1 µg/kg bw. Only parameters which affect C_{max} of DON in the intestinal plasma (A) and ileum lumen (B) with a normalized SC (absolute value) > 0.1 are shown. Parameters represent the following: BW: body weight human; VLc: fraction of liver; VSc: fraction of slowly perfused tissue; QSc; fraction of blood flow to slowly perfused tissue QIc: fraction of blood flow to the intestine tissue; PappDON: apparent permeability coefficients of DON; D: average diameter of intestine; kin2: transfer rate of DON from ileum to large intestine; VLM: human liver microsome protein yield; CLintDONc: unscaled DON clearance of liver microsome; RDON: blood/plasma ratio of DON; ksto: stomach emptying rate; L2: length of ileum; kin1: transfer rate of DON from jejunum to ileum.

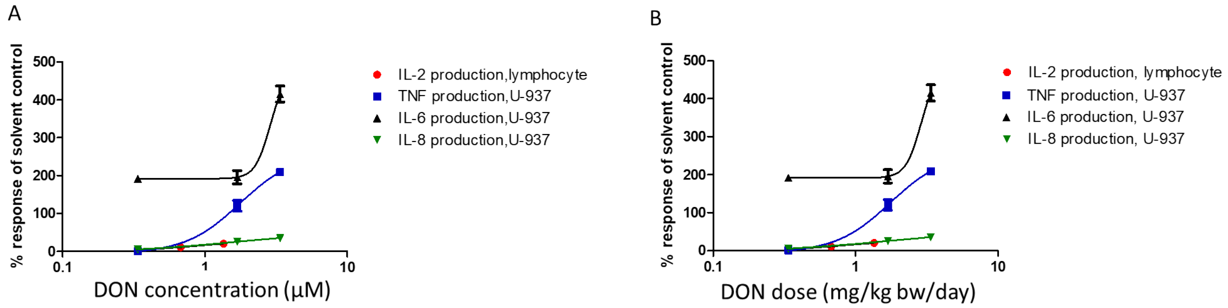

Fig. S3. Effect of DON on pro-inflammatory cytokines production. (A). Concentration-response curves of the effects of DON on IL-8, TNFα and IL-6 secretion in human U-937 macrophages [7] and on IL-2 secretion in human lymphocytes [8]. (B). Predicted *in vivo* dose-response curves of the induction effects of DON on IL-8, TNFα, IL-6 and IL-2 secretion in the intestine tissue using PBK modeling-based reverse dosimetry of the concentration-response curves in Fig. S3A.

Reference:

1. Brown, R.P., et al., *Physiological parameter values for physiologically based pharmacokinetic models.* Toxicology and industrial health, 1997. **13**(4): p. 407-484.
2. Vidal, A., et al., *Humans significantly metabolize and excrete the mycotoxin deoxynivalenol and its modified form deoxynivalenol-3-glucoside within 24 hours.* Scientific reports, 2018. **8**(1): p. 1-11.
3. Wang, X., et al., *Comprehensive dietary and internal exposure assessment of deoxynivalenol contamination in a high-risk area in China using duplicate diet studies and urinary biomarkers.* Food Control, 2021. **124**: p. 107830.
4. Warth, B., et al., *New insights into the human metabolism of the Fusarium mycotoxins deoxynivalenol and zearalenone.* Toxicology letters, 2013. **220**(1): p. 88-94.
5. Evans, M.V. and M.E. Andersen, *Sensitivity analysis of a physiological model for 2, 3, 7, 8-tetrachlorodibenzo-p-dioxin (TCDD): assessing the impact of specific model parameters on sequestration in liver and fat in the rat.* Toxicological sciences, 2000. **54**(1): p. 71-80.
6. Rietjens, I.M., J. Louisse, and A. Punt, *Tutorial on physiologically based kinetic modeling in molecular nutrition and food research.* Molecular nutrition & food research, 2011. **55**(6): p. 941-956.
7. Sugita-Konishi, Y. and J.J. Pestka, *Differential upregulation of TNF-α, IL-6, and IL-8 production by deoxynivalenol (vomitoxin) and other 8-ketotrichothecenes in a human macrophage model.* Journal of Toxicology and Environmental Health Part A, 2001. **64**(8): p. 619-636.
8. Meky, F., et al., *Deoxynivalenol-induced immunomodulation of human lymphocyte proliferation and cytokine production.* Food and chemical toxicology, 2001. **39**(8): p. 827-836.

Supplementary material 2

```
; Date: April 2022

; Purpose: General PBK Model deoxynivalenol (DON), built with in vitro and in silico derived parameter values

; Species: Human

; Compiled by: Jingxuan Wang

; Organisation: Wageningen University

;=====================================================================
; Physiological parameters
;=====================================================================
; Tissue volumes,
BW = 70             {Kg}        ; body weight human (variable, dependent on study)
VLc = 0.026                     ; fraction of liver tissue                          reference [1]
VRc = 0.055                     ; fraction of rapidly perfused tissue
      reference [1]
VSc = 0.603                     ; fraction of slowly perfused tissue                reference [1]
VFc = 0.214                     ; fraction of fat tissue                            reference [1]
VBc = 0.079                     ; fraction of blood                                 reference [1]
VIc = 0.009                     ; fraction of intestinal tissue                     reference [1]

VL = VLc*BW         {L or Kg}         ; volume of liver tissue (calculated)
VR = VRc*BW         {L or Kg}         ; volume of rapidly perfused tissue (calculated)
VS = VSc*BW         {L or Kg}         ; volume of slowly perfused tissue (calculated)
VF = VFc*BW         {L or Kg}         ; volume of fat tissue (calculated)
VB = VBc*BW         {L or Kg}         ; volume of blood (calculated)
VI = VIc*BW         {L or Kg}         ; volume of intestinal tissue (calculated)
VLU = 0.561         {L}               ; volume of large intestine content [2]
;---------------------------------------------------------------------------------------------------------------
; Blood flow rates
QC = 15*BW^0.74     {L/h}       ; cardiac output:15*BW^0.74                         reference [1]
QLc = 0.046                     ; fraction of blood flow to liver                   reference [1]
QRc = 0.473                     ; fraction of blood flow to rapidly perfused tissue reference [1]
QSc = 0.248                     ; fraction of blood flow to slowly perfused tissue  reference [1]
QFc = 0.052                     ; fraction of blood flow to fat                     reference [1]
QIc = 0.181                     ; fraction of blood flow to intestinal tissue       reference [1]

QL = QLc*QC         {L/h}             ; blood flow to liver tissue (calculated)
QR = QRc*QC         {L/h}             ; blood flow to rapidly perfused tissue (calculated)
QS = QSc*QC         {L/h}             ; blood flow to slowly perfused tissue (calculated)
QF = QFc*QC         {L/h}             ; blood flow to fat tissue (calculated)
QI = QIc*QC         {L/h}             ; blood flow to intestinal tissue (calculated)

;=====================================================================
; Physicochemical parameters
;=====================================================================
; partition coefficients, calculated using QPPR of DeJongh et al. [3]

LogDON = -0.71                          ; log Kow DON
RDON =   0.6523                         ; blood : plasma ratio
FuDON = 0.862                           ; fraction unbound plasma

PLDON = 0.76/RDON                       ; liver/blood partition coefficient
PRDON = 0.79/RDON                       ; rapidly perfused tissue/blood partition coefficient
PSDON = 0.64/RDON                       ; slowly perfused tissue/blood partition coefficient
PFDON = 0.18/RDON                       ; fat/blood partition coefficient
PIDON = 0.73/RDON                       ; intestinal tissue/blood partition coefficient
;---------------------------------------------------------------------------------------------------------------;
```

```
;Absorption/transfer rates:

;Stomach emptying rate
ksto = 1.99                                         ;stomach emptying rate (/h), reference [4]

;Intestinal absorption and transfer rates of DON
;PappCaco2DON = 3.3                                 ;( x10^-6 cm/sec), from Caco-2 reference [5]
;Log (Papp,in vivo) = 0.4926*Log(PappCaco2DON)-0.1454    ;( x10^-4 cm/sec),  in vivo reference [6]
;PappDONc=10^((0.4926*Log(PappCaco2DON)-0.1454))    ;( x10^-4 cm/sec)
PappDONc=1.2883
PappDON= PappDONc/10000*3600/10                     ;(dm/h)

D = 0.5            ;average diameter of small intestine or large intestine (dm) [7]
L1 = 30.0          ;length of jejunum (dm) [7]
L2 = 30.0          ;length of ileum (dm) [7]
L3 = 18.7          ;length of large intestine (dm) [7]

Vin1 = 3.14*(D/2)*(D/2)*L1          ;volume of jejunum lumen (L)
SAin1 = 3.14*D*L1                   ;surface area of jejunum lumen (dm2)
kab1 = PappDON*SAin1/Vin1           ;absorption rate constant of jejunum lumen (/hr)
kin1 = 2.17                         ;transfer rate to ileum lumen (/hr) [4]

Vin2 = 3.14*(D/2)*(D/2)*L2          ;volume of ileum lumen (L)
SAin2 = 3.14*D*L2                   ;surface area of ileum lumen (dm2)
kab2 = PappDON*SAin2/Vin2           ;absorption rate constant of ileum lumen (/hr)
kin2 = 0.25                         ;transfer rate of DON from ilem to large intestine (/hr) [4]

SAin3 = 3.14*D*L3                   ;surface area of large intestine (dm2)
kab3= PappDON*SAin3/VLU             ;absorption rate constant from the large intestine (/hr)

;Excretion rates
;Glomerular filtration
GFR=1.8                             ; (mL/min/kg bw), reference [8]
GF = GFR/1000*BW*60                 ; (L/hr), human glomerular filtration rate
;=====================================================================
; Kinetic parameters
;=====================================================================
; Clearance of DON in the liver

; scaling factors
VLM = 32         {mg microsomal protein/gram liver}      ; liver microsome protein yield     reference [9]
L=VLc*1000       {gram/kg BW}                            ; liver

CLintDONc=0.008      {ml/min/mg microsomal protein}      ;reference [10]
CLintDON= CLintDONc *VLM*L*BW*60/1000 {L/h/liver}

;=====================================================================
; Run settings
;=====================================================================

; molecular weight
MWDON = 296.3                       ; molecular weight DON

;---------------------------------------------------------------------------------------------------------
; oral dose
ODOSEmg =0.001                      {mg/kg bw}          ; oral dose, variable

ODOSEumol= ODOSEmg*1000/MWDON*BW    {μmol}              ; unit change to μmol
```

```
;-----------------------------------------------------------------------------------------------------------------
; time
Starttime = 0                                   {h}
Stoptime = 24                                   {h}                             ; variable
;=================================================================================
; Main model calculations/dynamics: DON
;=================================================================================
; Stomach
; Ast = amount in stomach
Ast' = -ksto*Ast
Init Ast = ODOSEumol
;-----------------------------------------------------------------------------------------------------------------
; small intestine lumen compartment
; intestines, divided in 2 compartments: jejunum and ileum
; Ain1 = Amount DON in jejunum compartment  (µmol)
Cin1 = Ain1/Vin1
Ain1' = ksto*Ast - kin1*Ain1- kab1*Ain1
Init Ain1 = 0
; Ain2 = Amount DON in ileum compartment  (µmol)
Cin2 = Ain2/Vin2
Ain2' = kin1*Ain1 - kin2*Ain2 - kab2*Ain2
Init Ain2 = 0
;-----------------------------------------------------------------------------------------------------------------
; large intestine lumen compartment
; ALIDON: amount of DON in large intestine lumen, {µmol}
    ALIDON' = kin2*Ain2 - kab3*ALIDON
    Init ALIDON = 0
    CLIDON= ALIDON/VLU
;-----------------------------------------------------------------------------------------------------------------
; intestinal tissue compartment
; AIDON: amount of DON in the intestinal tissue, {µmol}
    AIDON' = QI*(CB-CVIDON) + kab1*Ain1+ kab2*Ain2 + kab3* ALIDON
    Init AIDON = 0
    CIDON = AIDON/VI
        CVIDON = CIDON/PIDON
        CVIPDON = CVIDON/RDON
;-----------------------------------------------------------------------------------------------------------------
; liver compartment
; ALDON: amount of DON in liver, {µmol}
    ALDON' = QL*CB + QI*CVIDON - (QL+QI)*CVLDON - ALDONclear'
    Init ALDON=0
    CLDON = ALDON/VL
    CVLDON= CLDON/PLDON

        ALDONclear' = CLintDON * CVLDON
        Init ALDONclear =0

;-----------------------------------------------------------------------------------------------------------------
; fat compartment
; AF = amount of DON in fat tissue, {µmol}
    AF' = QF*(CB-CVF)
    Init AF = 0
    CF = AF/VF
    CVF = CF/PFDON

;-----------------------------------------------------------------------------------------------------------------
; rapidly perfused tissue
; AR = amount of DON in rapidly perfused tissue, {µmol}
    AR' = QR*(CB-CVR)
    Init AR = 0
```

```
        CR = AR/VR
        CVR = CR/PRDON

    ;------------------------------------------------------------------------------------------------------------------
    ; slowly perfused tissue
    ; AS = amount of DON in slowly perfused tissue, {µmol}
        AS' = QS*(CB-CVS)
        Init AS = 0
        CS = AS/VS
        CVS = CS/PSDON

    ;------------------------------------------------------------------------------------------------------------------
    ; blood compartment
    ; AB: amount of DON in blood, {µmol}
        AB' = (QL+QI)*CVLDON + QF*CVF+QS*CVS+QR*CVR – (QL+QI+QF+QS+QR)*CB -
GF*CB*FuDON
        Init AB = 0
        CB = AB/VB
        AUC'=AB
        Init AUC=0
    ;------------------------------------------------------------------------------------------------------------------
    ; urinary excretion, {µmol}
            Aur'= GF*CB*FuDON
        Init Aur = 0
    ;==================================================================
    ; Main model: mass balance calculation
    ;==================================================================
    Total = ODOSEumol
    Calculated = Ast + Ain1 + Ain2 + ALIDON + AIDON + ALDON + ALDONclear + AF + AR + AS + AB +
Aur

    ERROR=((Total-Calculated)/Total+1E-30)*100
    MASSBBAL=Total-Calculated + 1
```

References

1. Brown, R.P., et al., *Physiological parameter values for physiologically based pharmacokinetic models.* Toxicology and industrial health, 1997. **13**(4): p. 407-484.
2. Pritchard, S.E., et al., *Fasting and postprandial volumes of the undisturbed colon: normal values and changes in diarrhea‐predominant irritable bowel syndrome measured using serial MRI.* Neurogastroenterology & Motility, 2014. **26**(1): p. 124-130.
3. DeJongh, J., H.J. Verhaar, and J.L. Hermens, *A quantitative property-property relationship (QPPR) approach to estimate in vitro tissue-blood partition coefficients of organic chemicals in rats and humans.* Archives of Toxicology, 1997. **72**(1): p. 17-25.
4. Kimura, T. and K. Higaki, *Gastrointestinal transit and drug absorption.* Biological and Pharmaceutical Bulletin, 2002. **25**(2): p. 149-164.
5. Kadota, T., et al., *Comparative study of deoxynivalenol, 3-acetyldeoxynivalenol, and 15-acetyldeoxynivalenol on intestinal transport and IL-8 secretion in the human cell line Caco-2.* Toxicology in Vitro, 2013. **27**(6): p. 1888-1895.
6. Sun, D., et al., *Comparison of human duodenum and Caco-2 gene expression profiles for 12,000 gene sequences tags and correlation with permeability of 26 drugs.* Pharmaceutical research, 2002. **19**(10): p. 1400-1416.
7. Kararli, T.T., *Comparison of the gastrointestinal anatomy, physiology, and biochemistry of humans and commonly used laboratory animals.* Biopharmaceutics & drug disposition, 1995. **16**(5): p. 351-380.
8. Walton, K., J. Dorne, and A. Renwick, *Species-specific uncertainty factors for compounds eliminated principally by renal excretion in humans.* Food and chemical toxicology, 2004. **42**(2): p. 261-274.
9. Barter, Z.E., et al., *Scaling factors for the extrapolation of in vivo metabolic drug clearance from in vitro data: reaching a consensus on values of human micro-somal protein and hepatocellularity per gram of liver.* Current drug metabolism, 2007. **8**(1): p. 33-45.
10. Fæste, C.K., et al., *Prediction of deoxynivalenol toxicokinetics in humans by in vitro-to-in vivo extrapolation and allometric scaling of in vivo animal data.* Archives of Toxicology, 2018. **92**(7): p. 2195-2216.

Supplementary material 3

```
; Date: May 2022

; Purpose: General PBK Model GCDCA, built with in vitro and in silico derived parameter values
; Species: Human
; Compiled by: Jingxuan Wang and Véronique de Bruijn
; Organization: Wageningen University
;==================================================================================
;Physiological parameters
;==================================================================================
; tissue volumes
BW = 70 {Kg}            ; body weight human (variable, dependent on study)
VFc = 0.214             ; fraction of fat tissue                          reference [1]
VLc = 0.026             ; fraction of liver tissue                        reference [1]
VRc = 0.054             ; fraction of richly perfused tissue              reference [1]
VSc= 0.6033             ; fraction of slowly perfused tissue              reference [1]
VBc = 0.079             ; fraction of blood                               reference [1]
VIc = 0.009             ; fraction of intestinal tissue                   reference [1]
VGc= 0.0007             ; fraction of gallbladder tissue                  reference [2]

VF = VFc*BW             {L or Kg}      ; volume of fat tissue (calculated)
VL = VLc*BW             {L or Kg}      ; volume of liver tissue (calculated)
VR = VRc* BW            {L or Kg}      ; volume of  richly perfused tissue (calculated)
VS = VSc*BW             {L or Kg}      ; volume of  slowly perfused tissue (calculated)
VB = VBc* BW            {L or Kg}      ; volume of  blood (calculated)
VI = VIc*BW             {L or Kg}      ; volume of intestinal tissue (calculated)
VG= VGc*BW              {L or Kg}      ; volume of gall bladder tissue (calculated)
;----------------------------------------------------------------------------------
;blood flow rates
QC = 15*BW^0.74 {L/hr}  ; cardiac output                                        reference [1]
QFc = 0.052             ; fraction of blood flow to fat tissue                  reference [1]
QLc = 0.046             ; fraction of blood flow to liver (excluding portal vein)   reference [1]
QSc = 0.248             ; fraction of blood flow to slowly perfused tissue      reference [1]
QRc = 0.473             ; fraction of blood flow to richly perfused tissue      reference [1]
QIc = 0.181             ; fraction of blood flow to intestines                  reference [1]

QF = QFc*QC             {L/hr}    ; blood flow to fat tissue (calculated)
QL = QLc*QC             {L/hr}    ; blood flow to liver tissue (calculated)
QS = QSc*QC             {L/hr}    ; blood flow to  slowly perfused tissue (calculated)
QR = QRc*QC             {L/hr}    ; blood flow to richly perfused tissue (calculated)
QI = QIc*QC             {L/hr}    ; blood flow to intestines (calculated)
;==================================================================================
;Physicochemical parameters
;==================================================================================
;partition coefficients
logP =2.21              ; reference[3]
RGCDCA=0.55             ; blood:plasma ratio, 1-Hct, assumption

PF =0.05/RGCDCA         ; fat/blood partition coefficient
PL = 0.09/RGCDCA        ; liver/blood partition coefficient
PR = 0.125/RGCDCA       ; richly perfused tissue/blood partition coefficient
PS = 0.19/RGCDCA        ; richly perfused tissue/blood partition coefficient
PG=0.16/RGCDCA          ; gut/blood partition coefficient
;==================================================================================
;Kinetic parameters
;==================================================================================
;absorption rate constant from ileum lumen, inhibited by DON concentration in ileum lumen, experimental data
ka = -231.5 + (0.9222+231.5)/(1+10^((7.360-Cin2)*(-0.3966)))      {/hr}

; excretion through the colon.
Kf=0.05*ALu             {umol/h}       ;5% of bile acids escape ileum reabsorption [4].
Ks=Kf                   {umol/h}       ;de novo synthesis in liver equals excretion.

;biliary excretion from liver to bile canaliculi
VmaxBSEPc=5.848     {umol/min/mg BSEP}          ; reference [5]
```

```
KmBSEP = 4.3            {umol/L}                              ; reference [5]


MC=0                                  ; switch for Monte Carlo
aBSEPc=IF MC =1 THEN init(exp(NORMAL(-0.26, 0.403))) ELSE 0.839; BSEP protein abundance in pmol/10^6
hepatocytes, reference Burt (2016)
aBSEP= IF aBSEPc  > 0.23 AND aBSEPc < 2.58  THEN aBSEPc ELSE 0.00000001; BSEP protein abundance in pmol/10^6
hepatocytes, reference [6]

MWBSEP=140000                                                           ; BSEP is a 140 kDa protein, 140 000 g/mol
Hep=99              {10^6 hepatocytes/g liver}                          ; reference [7]
WL=20*BW            {g}                                                 ; reference [8]
SF=aBSEP*MWBSEP*Hep*WL*60*10^-9 {mg BSEP/entire lever}; scaling factor, calculated
VmaxBSEP=VmaxBSEPc*SF            {umol/h/entire liver}

;distribution of bile flow excreted from liver in the bile canaliculae
QIb = 0.5
; fraction of bile flow transported directly from liver to intestinal lumen via common bile duct reference:  Molino (1986)
QGb = 1- QIb                                         ; fraction of bile flow from liver stored in gall bladder, calculated

;systemic plasma concentration in fasting state
CBfs=2.4*sens                  {umol/L}                       ; reference [9]

;sensitivity individual
sens=1
;==========================================================================================
;Run settings
;==========================================================================================
Gdose =3020*sens{umol}                                        ; dose in full gallbladder [10]
;dosingperiod =if time < 12 OR time > 32 AND time <44  OR time >56 AND time <68 THEN  1 else 0 ; stop gallbladder
contractions during the night

dosingperiod =if time < 20 OR time > 32 AND time <44  OR time >56 AND time <68 THEN  1 else 0 ; stop gallbladder
contractions during the night

;time
Starttime = 8 ; in hr
Stoptime = 50; in hr
DTMIN=1E-6
DTMAX=1E-4
DTOUT=0.01
TOLERANCE=1E-12
Method Auto
;==========================================================================================
;Model calculations
;==========================================================================================
; gall bladder compartment
;AG = amount in the gallbladder, umol
;AG' = Change in amount in the gallbladder, umol/hr
AG'=-pulse(AG,0, 4)*dosingperiod + VmaxBSEP*CVL/(KmBSEP+ CVL)*QGb
Init AG = Gdose
;------------------------------------------------------------------------------------------
; liver compartment
;AL = Amount in liver tissue, umol
;AL' = Change in amount in liver tissue in time,  umol/hr
AL' =QL*CB + QI*CVI – (QL+QI)*CVL -VmaxBSEP*CVL/(KmBSEP+ CVL) + Ks
            CL = AL/VL
            CVL = CL/PL
Init AL=0
;------------------------------------------------------------------------------------------
; intestine compartment
;ALu= amount in ileum lumen, umol
ALu'=pulse(AG,0, 4)*dosingperiod+VmaxBSEP*CVL/(KmBSEP+ CVL)*QIb-Kf-ka*ALu
Init ALu=0

;AI' = amount GCDCA in the intestinal tissue remaining, umol
AI' = QI*(CB-CVI) + ka*ALu
```

```
Init AI = 0
CI=AI/VI
CVI=CI/PG

;ALCu=amount in ascending colon, umol
ALCu'= Kf
Init ALCu =0

;------------------------------------------------------------------------------------------------------------------------------------------
;fat compartment
;AF = Amount GCDCA in fat tissue, umol
            AF' = QF*(CB-CVF)
Init AF = 0
CF = AF/VF
            CVF = CF/PF
;------------------------------------------------------------------------------------------------------------------------------------------
;tissue compartment richly perfused tissue
;AR = Amount GCDCA in richly perfused tissue, umol
      AR' = QR*(CB-CVR)
      Init AR = 0
      CR = AR/VR
      CVR = CR/PR
;------------------------------------------------------------------------------------------------------------------------------------------
;tissue compartment slowly perfused tissue
;AS = Amount GCDCA in slowly perfused tissue, umol
      AS' = QS*(CB-CVS)
      Init AS = 0
      CS = AS/VS
      CVS = CS/PS
;------------------------------------------------------------------------------------------------------------------------------------------
; blood compartment
;AB = Amount GCDCA in blood (umol)
     AB' = QF*CVF + (QL+QI)*CVL  + QS*CVS + QR*CVR – (QF+QL+QS+QR+QI)*CB
     Init AB =0
     CB = AB/VB
     CBtot=CB/RGCDCA+CBfs                                   ; concentration GCDCA in plasma, umol/L
;------------------------------------------------------------------------------------------------------------------------------------------
; Mass balance calculations
Total =Gdose+Ks
Calculated = AL+ AS+ AR + AB + AG + AF + AI +Kf + ALu

ERROR=((Total-Calculated)/Total+1E-30)*100
MASSBBAL=Total-Calculated + 1



;Submodel deoxynivalenol (DON)
;==========================================================================================
;Physiological parameters
;==========================================================================================
; Tissue volumes,
VRDONc = (VRc + VGc)                    ; fraction of rapidly perfused tissue                    reference
[1]

VRDON = VRDONc*BW          {L or Kg}    ; volume of rapidly perfused tissue (calculated)
VLUDON = 0.561             {L}          ; volume of large intestine content (calculated)

;==========================================================================================
;Physicochemical parameters
;==========================================================================================
;partition coefficients
; partition coefficients, calculated using QPPR of DeJongh et al. [11]
LogDON = -0.71                          ; log Kow DON
RDON =   0.6523                         ; blood : plasma ratio
FuDON = 0.862                           ; fraction unbound plasma

PLDON = 0.76/RDON                       ; liver/blood partition coefficient
```

```
PRDON = 0.79/RDON                          ; rapidly perfused tissue/blood partition coefficient
PSDON = 0.64/RDON                          ; slowly perfused tissue/blood partition coefficient
PFDON = 0.18/RDON                          ; fat/blood partition coefficient
PIDON = 0.73/RDON                          ; intestinal tissue/blood partition coefficient
;=====================================================================================
;Kinetic parameters
;Absorption/transfer rates:

;Stomach emptying rate
ksto = 1.99                                          ;stomach emptying rate (/h), reference [12]

;Intestinal absorption and transfer rates of DON
;PappCaco2DON = 3.3                                  ;( x10^-6 cm/sec), from Caco-2 reference [13]
;Log (Papp,in vivo) = 0.4926*Log(PappCaco2DON)-0.1454  ;( x10^-4 cm/sec),  in vivo reference [14]
;PappDONc=10^((0.4926*Log(PappCaco2DON)-0.1454))     ;( x10^-4 cm/sec)
PappDONc=1.2883
PappDON= PappDONc/10000*3600/10                      ;(dm/h)

D = 0.5                                  ;average diameter of small intestine or large intestine (dm) [15]
L1 = 30.0                                ;length of jejunum (dm) [15]
L2 = 30.0                                ;length of ileum (dm) [15]
L3 = 18.7                                ;length of large intestine (dm) [15]

Vin1 = 3.14*(D/2)*(D/2)*L1                 ;volume of jejunum lumen (L)
SAin1 = 3.14*D*L1                          ;surface area of jejunum lumen (dm2)
kab1 = PappDON*SAin1/Vin1                  ;absorption rate constant of jejunum lumen (/hr)
kin1 = 2.17                                ;transfer rate to ileum lumen (/hr) [12]

Vin2 = 3.14*(D/2)*(D/2)*L2                 ;volume of ileum lumen (L)
SAin2 = 3.14*D*L2                          ;surface area of ileum lumen (dm2)
kab2 = PappDON*SAin2 /Vin2                 ;absorption rate constant of ileum lumen (/hr)
kin2 = 0.25                                ;transfer rate of DON from ilem to large intestine (/hr) [12]

SAin3 = 3.14*D*L3                          ;surface area of large intestine (dm2)
kab3= PappDON*SAin3/VLUDON                       ;absorption rate constant from the large intestine (/hr)

;Excretion rates
;Glomerular filtration
GFR=1.8                                    ; (mL/min/kg bw), reference [16]
GF = GFR/1000*BW*60                        ; (L/hr), human glomerular filtration rate
;-------------------------------------------------------------------------------------

; Clearance of DON in the liver

; scaling factors
VLM = 32          {mg microsomal protein/gram liver}        ; liver microsome protein yield      reference [7]
L=VLc*1000        {gram/kg BW}                              ; liver

CLintDONc=0.008      {ml/min/mg microsomal protein}         ;reference [17]
CLintDON= CLintDONc *VLM*L*BW*60/1000 {L/h/liver}
;=====================================================================================
;Run settings
;=====================================================================================
; molecular weight
MWDON = 296.3                        ; molecular weight DON

;-------------------------------------------------------------------------------------
; oral dose
ODOSEdaymg =0.001                          {mg/kg bw}           ; oral dose, variable
ODOSEmealmg=ODOSEdaymg/3                                        ; equally distribute in 3 meals
ODOSEumol= ODOSEmealmg*1000/MWDON*BW       {μmol}               ; unit change to μmol


;=====================================================================================
; Main model calculations/dynamics: DON
;=====================================================================================
; Stomach
```

```
; Ast = amount in stomach
Ast' = pulse(ODOSEumol, 0,4) *dosingperiod -ksto*Ast
Init Ast = 0
;--------------------------------------------------------------------------------------------------------------------------------------------------------
; small intestine lumen compartment
; intestines, divided in 2 compartments: jejunum and ileum
; Ain1 = Amount DON in jejunum compartment  (µmol)
Cin1 = Ain1/Vin1
Ain1' = ksto*Ast - kin1*Ain1- kab1*Ain1
Init Ain1 = 0
; Ain2 = Amount DON in ileum compartment  (µmol)
Cin2 = Ain2/Vin2
Ain2' = kin1*Ain1 - kin2*Ain2 - kab2*Ain2
Init Ain2 = 0
;--------------------------------------------------------------------------------------------------------------------------------------------------------
; large intestine lumen compartment
; ALIDON: amount of DON in large intestine lumen, {µmol}
            ALIDON' = kin2*Ain2 - kab3*ALIDON
            Init ALIDON = 0
            CLIDON= ALIDON/VLUDON
;--------------------------------------------------------------------------------------------------------------------------------------------------------
; Intestinal tissue compartment
; AIDON: amount of DON in the intestinal tissue, {µmol}
            AIDON' = QI*(CBDON-CVIDON) + kab1*Ain1+ kab2*Ain2 + kab3*ALIDON
            Init AIDON = 0
            CIDON = AIDON/VI
            CVIDON = CIDON/PIDON
            CVIPDON = CVIDON/RDON
;--------------------------------------------------------------------------------------------------------------------------------------------------------
; liver compartment
; ALDON: amount of DON in liver, {µmol}
            ALDON' = QL*CB + QI*CVIDON - (QL+QI)*CVLDON - ALDONclear'
            Init ALDON=0
            CLDON = ALDON/VL
            CVLDON= CLDON/PLDON

            ALDONclear' = CLintDON * CVLDON
            Init ALDONclear =0

;--------------------------------------------------------------------------------------------------------------------------------------------------------
; fat compartment
; AFDON = amount of DON in fat tissue, {µmol}
AFDON' = QF*(CBDON-CVFDON)
Init AFDON = 0
CFDON = AFDON/VF
CVFDON = CFDON/PFDON

;--------------------------------------------------------------------------------------------------------------------------------------------------------
; rapidly perfused tissue
; ARDON = amount of DON in rapidly perfused tissue, {µmol}
ARDON' = QR*(CBDON-CVRDON)
Init ARDON = 0
CRDON = ARDON/VRDON
CVRDON = CRDON/PRDON

;--------------------------------------------------------------------------------------------------------------------------------------------------------
; slowly perfused tissue
; ASDON = amount of DONdzein in slowly perfused tissue, {µmol}
ASDON' = QS*(CBDON-CVSDON)
Init ASDON = 0
CSDON = ASDON/VS
CVSDON = CSDON/PSDON

;--------------------------------------------------------------------------------------------------------------------------------------------------------
; blood compartment
; ABDON: amount of DON in blood, {µmol}
ABDON' = (QL+QI)*CVLDON + QF*CVFDON+QS*CVSDON+QR*CVRDON - QC*CBDON - GF*CBDON*FuDON
```

```
Init ABDON = 0
CBDON = ABDON/VB
AUC'=ABDON
Init AUC=0
;--------------------------------------------------------------------------------------------------------------------------------------------------
; urinary excretion, {µmol}
	Aur'= GF*CBDON*FuDON
	Init Aur = 0
;==========================================================================================
; Main model: mass balance calculation
;==================================================================== ===================
TotalDON' = pulse(ODOSEumol, 0,4) *dosingperiod
Init TotalDON = 1E-50
CalculatedDON = Ast + Ain1 + Ain2 + ALIDON + AIDON + ALDON + ALDONclear + AFDON + ARDON + ASDON +
ABDON + Aur

ERRORDON=((TotalDON-CalculatedDON)/Total+1E-30)*100
MASSBBALDON=TotalDON-CalculatedDON + 1
```

Reference:

1. Brown, R.P., et al., *Physiological parameter values for physiologically based pharmacokinetic models.* Toxicology and industrial health, 1997. **13**(4): p. 407-484.
2. Van Erpecum, K.J., et al., *Fasting gallbladder volume, postprandial emptying and cholecystokinin release in gallstone patients and normal subjects.* Journal of hepatology, 1992. **14**(2-3): p. 194-202.
3. Roda, A., et al., *Bile acid structure-activity relationship: evaluation of bile acid lipophilicity using 1-octanol/water partition coefficient and reverse phase HPLC.* Journal of lipid research, 1990. **31**(8): p. 1433-1443.
4. Voronova, V., et al., *A physiology-based model of bile acid distribution and metabolism under healthy and pathologic conditions in human beings.* Cellular and Molecular Gastroenterology and Hepatology, 2020. **10**(1): p. 149-170.
5. Kis, E., et al., *Effect of membrane cholesterol on BSEP/Bsep activity: species specificity studies for substrates and inhibitors.* Drug metabolism and disposition, 2009. **37**(9): p. 1878-1886.
6. Burt, H.J., et al., *Abundance of hepatic transporters in Caucasians: a meta-analysis.* Drug metabolism and disposition, 2016. **44**(10): p. 1550-1561.
7. Barter, Z.E., et al., *Scaling factors for the extrapolation of in vivo metabolic drug clearance from in vitro data: reaching a consensus on values of human micro-somal protein and hepatocellularity per gram of liver.* Current drug metabolism, 2007. **8**(1): p. 33-45.
8. Soars, M., B. Burchell, and R. Riley, *In vitro analysis of human drug glucuronidation and prediction of in vivo metabolic clearance.* Journal of Pharmacology and Experimental Therapeutics, 2002. **301**(1): p. 382-390.
9. García-Cañaveras, J.C., et al., *Targeted profiling of circulating and hepatic bile acids in human, mouse, and rat using a UPLC-MRM-MS-validated method.* Journal of lipid research, 2012. **53**(10): p. 2231-2241.
10. Sips, F.L., et al., *In silico analysis identifies intestinal transit as a key determinant of systemic bile acid metabolism.* Frontiers in physiology, 2018. **9**: p. 631.
11. DeJongh, J., H.J. Verhaar, and J.L. Hermens, *A quantitative property-property relationship (QPPR) approach to estimate in vitro tissue-blood partition coefficients of organic chemicals in rats and humans.* Archives of Toxicology, 1997. **72**(1): p. 17-25.
12. Kimura, T. and K. Higaki, *Gastrointestinal transit and drug absorption.* Biological and Pharmaceutical Bulletin, 2002. **25**(2): p. 149-164.
13. Kadota, T., et al., *Comparative study of deoxynivalenol, 3-acetyldeoxynivalenol, and 15-acetyldeoxynivalenol on intestinal transport and IL-8 secretion in the human cell line Caco-2.* Toxicology in Vitro, 2013. **27**(6): p. 1888-1895.
14. Sun, D., et al., *Comparison of human duodenum and Caco-2 gene expression profiles for 12,000 gene sequences tags and correlation with permeability of 26 drugs.* Pharmaceutical research, 2002. **19**(10): p. 1400-1416.
15. Kararli, T.T., *Comparison of the gastrointestinal anatomy, physiology, and biochemistry of humans and commonly used laboratory animals.* Biopharmaceutics & drug disposition, 1995. **16**(5): p. 351-380.
16. Walton, K., J. Dorne, and A. Renwick, *Species-specific uncertainty factors for compounds eliminated principally by renal excretion in humans.* Food and chemical toxicology, 2004. **42**(2): p. 261-274.
17. Fæste, C.K., et al., *Prediction of deoxynivalenol toxicokinetics in humans by in vitro-to-in vivo extrapolation and allometric scaling of in vivo animal data.* Archives of Toxicology, 2018. **92**(7): p. 2195-2216.
